# An updated checklist and key to the open-panicled species of *Poa* L. (Poaceae) in Peru including three new species, *Poa ramoniana*, *Poa tayacajaensis*, and *Poa urubambensis*

**DOI:** 10.3897/phytokeys.65.7024

**Published:** 2016-06-30

**Authors:** Steven P. Sylvester, Robert J. Soreng, Paul M. Peterson, Mitsy D.P.V. Sylvester

**Affiliations:** 1 Institute of Systematic and Evolutionary Botany, University of Zurich, Zollikerstrasse 107, 8008 Zürich, Switzerland; 2 Department of Geography, Philipps-Universität Marburg, Deutschhausstraße 10, D-35032 Marburg, Germany; 3 Department of Botany, National Museum of Natural History, Smithsonian Institution Washington, DC, 20013-7012, USA; 4 Universidad Nacional de San Antonio Abad del Cusco, Avenida de la Cultura 733, Cusco, Perú

**Keywords:** Checklist, Gramineae, new species, Peru, Poa, Pooideae, Polylepis forest, Pooideae, Puna grassland, grass taxonomy

## Abstract

We provide an updated checklist and key to the 30 *Poa* species with open panicles from Peru which includes previously circumscribed *Dissanthelium* and *Aphanelytrum* species, new taxon records, and three undescribed species. *Poa compressa*, *Poa grisebachii*, and *Poa leioclada* are recorded from Peru for the first time. A number of species are placed in synonymy: *Poa carazensis*, *Poa ferreyrae* and *Poa tovarii* are synonymized under the name *Poa fibrifera*; *Poa adusta* (tentatively) and *Poa pilgeri* are synonymized under *Poa candamoana*; *Poa superata* is synonymized under *Poa grisebachii*; and *Poa paramoensis* is synonymized under *Poa huancavelicae*. Included within this treatment are three new species, *Poa ramoniana*, *Poa tayacajaensis* and *Poa urubambensis*, which are described and illustrated. *Poa ramoniana*, found growing near lakes in high elevation Puna grasslands of Junín, is similar to a small form of *Poa glaberrima*, but differs in having rhizomes and growing to only 5 cm tall. *Poa tayacajaensis*, found from shrublands on Andean slopes of Huancavelica and Huánuco, bears similarities to *Poa aequatoriensis* but differs in having shorter lemmas which are pubescent between the veins, densely scabrous sheaths with smooth, glabrous throats, and shorter ligules. *Poa urubambensis*, a common element of the undisturbed *Polylepis* forest understory of the Cordillera Urubamba, Cusco, is distinct from all other members of open-panicled *Poa*’s by having glabrous lemmas with a smooth and glabrous callus, and notably small anthers. The type material for the name *Poa adusta* is discussed and a lectotype is selected.

## Abstract (es)

Aportamos una lista actualizada y una clave para las 30 especies de *Poa* con panículas abiertas de Perú que incluye las especies de *Dissanthelium* y *Aphanelytrum* anteriormente circunscritas, nuevos registros de taxones y tres especies no descritas. *Poa compressa*, *Poa grisebachii*, y *Poa leioclada* se registran para Perú por primera vez. Un número de especies son sinonimizadas: *Poa carazensis*, *Poa ferreyrae* y *Poa tovarii* son sinonimizadas bajo el nombre de *Poa fibrifera*; *Poa adusta* (tentativamente) y *Poa pilgeri* son sinonimizadas bajo el nombre de *Poa candamoana*; *Poa superata* es sinonimizada bajo *Poa grisebachii*; y *Poa paramoensis* es sinonimizada bajo *Poa huancavelicae*. Dentro de este tratamiento se incluyen dos especies nuevas, *Poa ramoniana*, *Poa tayacajaensis* y *Poa urubambensis* que a continuación se describen e ilustran. *Poa ramoniana*, que se encuentra creciendo en pastizales de alta elevación cercanos a lagos en la Puna de Junín, es similar a la forma pequeña de *Poa glaberrima*, pero se diferencia por tener rizomas y crecer hasta sólo 5 cm de altura. *Poa tayacajaensis*, que se encuentra en matorrales de las laderas andinas de Huancavelica y Huánuco, tiene un parecido a *Poa aequatoriensis*, pero se diferencia por tener lemas cortas que son pubescentes entre las venas, vainas densamente escabrosas con suaves gargantas glabras, y lígulas cortas. *Poa urubambensis*, un elemento común de sotobosque no perturbado de los bosques de *Polylepis* de la Cordillera Urubamba, Cusco, se distingue de todos los otros miembros de *Poa* con panícula-abierta por tener lemas glabras con un callo suave y glabro, y sus notables anteras pequeñas. El material tipo del nombre *Poa adusta* es discutido y un lectotipo es seleccionado.

## Introduction

The genus *Poa* L. is the largest genus of the Poaceae, containing over 500 species with a large distribution across temperate areas of the globe (Soreng et al. 2003, 2010, 2015, 2016). The first taxonomic treatment of *Poa* from Peru comes from Hitchcock’s (1927) ‘The grasses of Ecuador, Peru and Bolivia’ in which he mentions 17 species. Standley (1936), in the Flora of Peru series, produced the first treatment of purely Peruvian grasses in which he added *Poa aequatoriensis* to the country records, raising the total number of Peruvian *Poa* to 18. Following this, the renowned Peruvian agrostologist, Óscar Tovar Serpa, began his life-long work on Peruvian grasses producing a number of publications related to *Poa* (Tovar 1965, 1974, 1984, 1986) until his largest cumulatory work in 1993 where he provided a concise treatment of all grasses from Peru (Tovar 1993).

Tovar’s (1993) most up-to-date taxonomic treatment, and the Checklist for Peru (Brako and Zarucchi 1993) that was published in the same year, considered the country to have either 40 or 41 species of *Poa*, respectively. This number has since varied due to taxonomic revision placing certain names in synonymy (Soreng et al. 2003, 2016), and discoveries of undescribed species (Negritto and Antón 2006; Davidse et al. 2010; Soreng and Peterson 2010; Peterson and Soreng 2016). Additionally, DNA studies (Gillespie et al. 2007, 2008; Refulio-Rodríguez et al. 2012; Peterson and Soreng 2016) found four small, mostly closed-panicled, genera to be nested within Poa subg. Poa supersect. Homalopoa (Dumort.) Soreng & L.Gillespie. Species of *Anthochloa* Nees & Meyen, *Tovarochloa* T.D. Macfarl. & P. But, and *Dissanthelium* Trin., were thus transferred to *Poa* by Gillespie et al. (2007) and Refulio-Rodríguez et al. (2012), while species of *Aphanelytrum* (Hack.) Hack. were recently transferred to Poa by Peterson and Soreng (2016).

To this point, 51 species had been accepted in *Poa* for Peru (Soreng et al. 2016), four of which are considered to be exotic (*Poa annua* L., *Poa infirma* Kunth, *Poa pratensis* L. [subsp. *alpigena* (Lindm.) Hiitonen is indigenous in North America and Patagonia], *Poa trivialis* L.). Of these 51 species, 32 have open-panicled inflorescences. Panicle characteristics are good for separating Peruvian *Poa* into two distinct groups. All taxonomic treatments of *Poa* in Peru (Hitchcock 1927; Standley 1936; Tovar 1993), Bolivia (Renvoize 1998) and Ecuador (Hjorth 1991) have provided diagnostic keys that, in the first couplet, separate species into those with a congested spike-like panicle, with panicle branches appressed, and those with an open-panicled inflorescence, the branches spreading. We believe this artificial character to be convenient and reliable in separating Peruvian *Poa*.

Our objective is to provide an up-to-date summary of the open-panicled species of *Poa* in Peru including locality information using verified specimens and discussion of nomenclatural and taxonomic attributes, with the new species, *Poa ramoniana*, *Poa tayacajaensis* and *Poa urubambensis*, being described and illustrated. Two keys are provided to aid with identification of the open-panicled *Poa*. The main key first uses anther length to separate taxa while the Suppl. material 1 first uses lemma indumentum. We hope users of our keys will have a better chance of accurately identifying Peruvian specimens of *Poa*.

## Materials and methods

In this treatment, glabrous means without pubescence (in the sense of slender, relatively soft hairs). Smooth indicates no prickle-hairs with broad bases and/or hooked or pointed apices (i.e., pubescence can occur on a smooth surface, and a rough or scabrous surface can be glabrous). Specimen localities in the checklist are cited by political region (also historically called ‘departamento’) (capital letters) and then province. Only herbaria where specimens have been checked and verified by the authors have been cited (acronyms following Thiers, continuously updated): Mainly MO (material on loan to US, c. 240 collections of *Poa* from Peru [*Peterson* duplicates excepted], and many more from across South America), and US, but USM specimens and types were examined in-situ by RJS (in 2006 and 2007), and CUZ and Z specimens were examined by SPS. Almost all *P.M. Peterson Poa* collections (first set at US, c. 460 collections from Peru) are duplicated at USM, although the USM duplicates have not been re-checked for this paper. Excluded species are discussed at the end of the checklist.

## Results

Of the 32 species of *Poa* with open-panicles previously recognized in Peru (Soreng et al. 2016), we consider three of these species records to be erroneous and have placed five species names in synonymy. Following the discovery of three undescribed species and three new country records, we now recognize 30 species of *Poa* with open-panicles in Peru.

The new species, *Poa urubambensis*, was found in remote areas of the Cordillera Urubamba, southern Peru, during recent fieldwork by the first author. While reviewing Peruvian specimens of open-panicled *Poa* from collections in the United States National Herbarium and Missouri Botanical Garden, a further two undescribed species, *Poa ramoniana* and *Poa tayacajaensis*, were discovered. *Poa ramoniana* was discovered from collections by eminent Peruvian botanist, Ramón Alejandro Ferreyra, from Junín. *Poa tayacajaensis* was discovered from collections by the renowned Peruvian agrostologist, Óscar Tovar Serpa, from the province of Tayacaja, Huancavelica.

We report new species records for: Poa cf. leioclada Hack., previously considered endemic to Ecuador (León Yánez et al. 2011), and *Poa grisebachii* R.E. Fr., previously considered endemic to Argentina (Giussani et al. 2012). *Poa compressa* L., originating from Europe, is also reported for the first time. Three species: *Poa carazensis* Pilg., *Poa ferreyrae* Tovar, and *Poa tovarii* Soreng, appear to be morphologically indistinct from *Poa fibrifera* Pilg. and have been placed under that name. *Poa adusta* J. Presl, known only from the type collection, and the recently described *Poa pilgeri* Negritto and Antón (2006: 87) are synonymized under *Poa candamoana* Pilg. Specimens of *Poa superata* Hack., previously known only from Argentina and Chile, have been collected from Peru and were determined to be morphologically indistinct from *Poa grisebachii* R.E. Fr. *Poa paramoensis* Lægaard, previously considered endemic to Ecuador (Lægaard 1998), has been found to be morphologically indistinct from *Poa huancavelicae* Tovar. Reports of *Poa lilloi* Hack. and *Poa supina* Schrad. for Peru are considered erroneous, and *Poa bromoides* Vahl (accepted by Brako and Zarucchi 1993) is currently accepted as *Eragrostis bromoides* (Vahl) Steud. by Soreng et al. (2016). These species were removed from the checklist.

### Key to the open-panicled species of *Poa* in Peru

**Table:** 

1	Lemmas with bifid apexes that are mucronate to short-awned; spikelets glabrous, with long rachillas 1.2–4.2 mm long; glumes short, less than ½ the length of the florets, sometimes absent	**2**
–	Lemmas with obtuse to acute apexes, never bifid, mucronate or short awned; spikelets usually with some form of indumentum, less often glabrous, with short rachillas rarely longer than 1.2 mm; glumes less than half to equaling the length of the spikelet, never absent	**3**
2 (1)	Glumes veinless, 0.1−0.5 (–0.7) mm long, minute or absent; plants straggling and stooling; culms 30–80(–100) cm tall	***Poa hitchcockiana***
–	Glumes veined, 1−2 mm long, lower glume 1-veined, upper glume 3- or 4-veined; plants caespitose; culms 14−24 cm tall	***Poa sanchez-vegae***
3 (1)	Glumes exceeding the florets; spikelets 2-flowered; lemmas 3 (rarely 5)-veined, glabrous, smooth or scaberulous; low tufted (sometimes rhizomatous in *Poa trollii*) perennial plants mostly less than 10 cm tall; panicles 1–3.3 cm long	**4**
–	Glumes shorter than the proximal floret; spikelets 2–4(–6)-flowered; lemmas 5(or 7)-veined, glabrous or pubescent, smooth or variously scabrous; annual or perennial plants of various habits, ranging mostly from 10–120 cm tall; panicles longer (sometimes short in the annuals)	**6**
4 (3)	Anthers 2–2.2 mm long in staminate specimens, vestigial in pistillate specimens; lemmas smooth throughout; plants sometimes rhizomatous	***Poa trollii***
–	Anthers <1 mm long in lower floret of spikelets, sometimes vestigial in upper floret; lemmas scaberulous, at least on the keels (rarely smooth in *Poa calycina*); plants densely tufted	**5**
5 (4)	Leaf blade abaxial surface shiny with veins indistinct	***Poa calycina***
–	Leaf blade abaxial surface dull with veins apparent	***Poa swallenii***
6 (3)	Longest anthers of proximal florets 0.2–1.5 mm long	**7**
–	Longest anthers of proximal florets (1.2–)1.6–3.2 mm long	**16**
7 (6)	Plants annual; palea keels distinctly pubescent in part (very rarely glabrous) always without any hooks; callus glabrous	**8**
–	Plants perennial; palea keels glabrous or pubescent in part, but always scabrous in part; callus glabrous or webbed, i.e. with long silky hairs (sometimes sparse) emerging from below the lemma keel (at least of the lower florets)	**9**
8 (7)	Anthers 0.2–0.5(–0.6) mm long; panicle branches ascending, spikelets usually crowded; foliage light green; plants ephemeral	***Poa infirma***
–	Anthers 0.6–1 mm long; panicle branches ascending to spreading, spikelets loosely arranged; foliage usually darker green; plants infrequently persisting for more than one season	***Poa annua***
9 (7)	Culm nodes strongly compressed, lower culm nodes exposed; culms wiry; plants strongly rhizomatous with isolated shoots; all florets of spikelets hermaphroditic (sometimes anthers aborted late in development)	***Poa compressa***
–	Combination of characters not as above; culm nodes terete or slightly compressed, lower culm nodes usually held within sheaths; culms varying from wiry to robust; plants tufted or rhizomatous; all florets of spikelets hermaphroditic, or upper floret(s) within spikelets sometimes pistillate, with rudimentary stamens (i.e. gynomonoecious)	**10**
10 (9)	Callus glabrous	***Poa urubambensis***
–	Callus webbed, i.e. with long silky hairs (sometimes sparse) emerging from below the lemma keel (at least of the lower florets)	**11**
11 (10)	Leaf blades filiform or slightly broader, involute or subinvolute towards the base, 0.5–2 mm wide when expanded; lower lemma 3–4 (5?) mm long, glabrous; web only (web sometimes v. short and sparse, and present on basal florets only)	***Poa pauciflora***
–	Leaf blades flat or folded, usually >2 mm wide when expanded; lower lemma 2.3–6 mm long, variously glabrous or pubescent	**12**
12 (11)	Upper ligules 0.9–2(–3) mm long, truncate; plants distinctly rhizomatous; lower sheaths smooth, sometimes lightly pubescent; lower lemma keel and marginal veins distinctly pubescent; spikelets with 2–6 florets; all florets of spikelets hermaphroditic (sometimes anthers aborted late in development)	***Poa pratensis***
–	Combination of characters not as above; upper ligules 0.2–10 mm long, acute or rarely truncate; plants tufted (weakly rhizomatous in *Poa huancavelicae*); lower sheaths smooth to densely scabrous; lower lemma keel and marginal veins varying in indumentum from glabrous to short pubescent; spikelets with 2–3 florets; upper floret within spikelets sometimes pistillate, with rudimentary stamens	**13**
13 (12)	Leaf blades folded, apex prominently naviculate (prow-tipped); plants weakly rhizomatous; lemma keels and marginal veins smooth or scaberulous, glabrous; callus webbing the only indumentum present in the spikelet	***Poa huancavelicae***
–	Leaf blades flat, apex not, or not prominently, naviculate; plants tufted, occasionally stooling and rooting at nodes; lemma keels short pubescent in the lower ½, sometimes sparingly so on the marginal veins near the base (rarely glabrous in *Poa aequatoriensis*); spikelet indumentum consisting of lemma pubescence (as mentioned above) and callus webbing	**14**
14 (13)	Spikelet proximal lemmas pubescent on keel, lateral, and marginal veins; distal lemmas pubescent between the veins; sheaths densely scabrous; ligules 2–3.5 mm long	***Poa tayacajaensis***
–	Combination of characters not as above; spikelet proximal lemmas glabrous or sparingly pubescent on the keel, and sometimes marginal veins; distal lemmas often glabrous throughout; sheaths smooth to densely scabrous; ligules 1–10 mm long	**15**
15 (14)	Lower culm sheaths usually puberulent in the throat margins and/or along the collar margins, surfaces smooth to lightly scabrous; upper culm leaf ligules 1–5(–7) mm long, abaxially puberulous or scabrous; lowest floret of spikelets hermaphroditic, upper florets commonly pistillate; spikelets 3.5–5 mm long; lower lemma 3.6–4 mm long, intermediate veins faint to moderately pronounced; palea keels usually finely scabrous to some degree; anthers mostly 0.6–1.5 mm long	***Poa aequatoriensis***
–	Lower culm sheaths glabrous in the throat margins, surfaces nearly smooth to densely scabrous; upper culm leaf ligules 4–10 mm long, abaxially smooth or faintly scabrous; all florets of spikelets hermaphroditic; spikelets 2.3–3.5(–4) mm long; lower lemma 2.3–3(–3.5) mm long, intermediate veins distinctly pronounced; palea keels usually muriculate, sometimes minutely scabrous; anthers (1) 1.3–1.6 (1.8) mm long	***Poa trivialis***
16 (6)	Lemmas glabrous, smooth or scabrous (rarely sericeous at the base in *Poa ramifera*); callus glabrous	**17**
–	Lemmas, at least of the upper florets, pubescent or villous in their lower half (rarely scabrous-pubescent in *Poa kurtzii*), or glabrous but then callus webbed, i.e. with long silky hairs emerging from below the lemma keel; callus glabrous or webbed	**26**
17 (16)	Culms erect, aerially branching well up the culm with lateral shoots that persist and flower in subsequent seasons	***Poa ramifera***
–	Culms not branching, or branching only near the base, or from decumbent culms	**18**
18 (17)	Summit of sheathes with prominent triangular auricles; spikelets 4–6-flowered	***Poa auriculata***
–	Sheathes without auricles; spikelets 2–6-flowered	**19**
19 (18)	Lemmas surface completely smooth (sometimes distally obscurely to sparsely scaberulous in *Poa ramoniana* but then plants 4–6 cm tall and ligules <1 mm long)	**20**
–	Lemmas slightly to strikingly scabrous between and on veins	**23**
20 (19)	Plants 4–6 cm tall; rhizomatous; ligules <1 mm long	***Poa ramoniana***
–	Plants >10 cm tall; tufted or, if rhizomatous >100 cm tall; ligules 2–7 mm long	**21**
21 (20)	Leaf blades smooth throughout, (2–)4–9 cm long, 1–2(–2.5) mm wide when blade flattened	***Poa glaberrima***
–	Leaf blades densely scabrous throughout, 8–40 cm long, 3–10 mm wide when blade flattened	**22**
22 (21)	Leaf blades conspicuously folded; plants 25–35 cm tall, tufted	***Poa gilgiana***
–	Leaf blades flat; plants 100–150 cm tall, rhizomatous	***Poa ayacuchensis***
23 (19)	Leaf blades involute or the margins distinctly involute (rarely simply folded in *Poa kurtzii*), densely scabrous (at least abaxially), firm to rigid; plants tufted; ligules (2.5–)5–15 mm long	**24**
–	Leaf blades flat or folded, margins rarely distinctly involute, glabrous or lightly scabrous, lax or firm; plants erect (*Poa fibrifera*) or rhizomatous (*Poa oscariana*), sometimes tufted; ligules 1–7(–9) mm long	**25**
24 (23)	Ligules 8–15 mm long; panicles narrowly ovate, panicle branches ascending and subappressed, panicles included in the sheaths; lemmas scabrous	***Poa pearsonii***
–	Ligules (2.5–)5–8 mm long; panicles amply ovate, panicle branches patent or reflexed, panicles exerted; lemmas scabrous or scabrous-pilose	***Poa kurtzii***
25 (23)	Lower leaf sheaths often fibrous; ligules 1.5–5(–6) mm long; blades lax; spikelets 3–5-flowered, 5–9 mm long; anthers 2.4–3.5 mm long; rachilla internodes well exposed	***Poa fibrifera***
–	Lower leaf sheaths not fibrous; ligules 6–9 mm long; blades somewhat firm; spikelets 2–3-flowered, 4.5–5 mm long; anthers 1.8–2 mm long; rachilla internodes short (compare with *Poa gilgiana*)	***Poa oscariana***
26 (16)	Callus glabrous; lemmas (at least the distal ones within a spikelet) softly villous-pubescent in their lower half	**27**
–	Callus webbed, i.e. with long silky hairs (sometimes sparse) emerging from below the lemma keel (at least of the lower florets); lemmas glabrous or distinctly to sparsely villous or serious pubescent along the keel and marginal veins only	**30**
27 (26)	Plants (30–)60–150 cm tall; leaf blades flat, sometimes folded towards their apices, usually more than 3 and up to 10 mm wide; inflorescence branches commonly verticillate; plants (sub-)rhizomatous with extravaginal shoots (if blades rather firm and folded but broad as in *Poa horridula*, compare with *Poa gilgiana*, possibly hybrids)	***Poa horridula***
–	Plants usually <35 cm tall; leaf blades involute to narrowly convolute and 0.5–2 mm wide, or flat to folded and 1–5 mm wide in *Poa grisebachii*; inflorescence branches solitary or paired (often 3 branches in basal nodes of *Poa grisebachii*); plants usually with only intravaginal shoots	**28**
28 (27)	Leaf blade abaxial surface densely scabrous; ligules (2.5–)5–8 mm long, acute; lemmas scabrous-pilose; plants of semi-arid habitats	***Poa kurtzii***
–	Leaf blade abaxial surface glabrous to scaberulous with prickles or hooks usually restricted to the leaf margin; ligules 0.5–3 mm long, truncate; lemmas pilose-villous towards base; plants of mesic or more arid habitats	**29**
29 (28)	Leaf blades usually involute, apex narrowly but abruptly naviculate (prow-tipped); spikelets usually 3-flowered, (2.8–)4.3–5.5 mm long; culm basal sheath bases slightly inflated, shiny, and tough; plants of more mesic Puna, mostly 3700–4500 m	***Poa candamoana***
–	Leaf blades flat or folded, somewhat lax, apex often tapered to a long slender point; spikelets 3–6-flowered, (5–)6–7.2 mm long; culm basal sheaths not as above; plants of more arid zones between 3000 and 4000 m (appears to hybridize with *Poa kurtzii* where the two overlap)	***Poa grisebachii***
30 (26)	Leaf blades filiform or slightly broader, involute or sub-involute towards the base, 0.5–2 mm wide when expanded; lower lemma 3–4 (5?) mm long, glabrous; web only (web sometimes v. short and sparse, and present on basal florets only)	***Poa pauciflora***
–	Leaf blades flat or folded, usually >2 mm wide when expanded; lower lemma 2.3–6 mm long, variously glabrous or pubescent	**31**
31 (30)	Basal sheaths glabrous and densely scabrous; lemmas smooth (or lightly scabrous near the apex), glabrous; web only	***Poa scabrivaginata***
–	Basal sheaths glabrous or lightly pubescent, smooth or lightly scabrous, or if densely scabrous then lemmas pubescent at least on the keel; lemmas smooth or scabrous, glabrous or pubescent in part	**32**
32 (31)	Upper ligules 0.9–2(–3) mm long, truncate; plants distinctly rhizomatous; lower sheaths smooth, sometimes lightly pubescent; lower lemma keel and marginal veins distinctly pubescent; spikelets with 2–6 florets; all florets of spikelets hermaphroditic (sometimes anthers aborted late in development)	***Poa pratensis***
–	Combination of characters not as above; upper ligules 0.2–10 mm long, acute or rarely truncate; plants tufted (usually with short rhizomatous shoots in *Poa huancavelicae* and prominent sub-rhizomatous extravaginal shoots present in *Poa leioclada*); lower sheaths smooth to densely scabrous; spikelets with 2–3(–4) florets; upper floret within spikelets hermaphroditic (i.e. *Poa trivialis*) or commonly pistillate, with rudimentary stamens (i.e. *Poa huancavelicae*, Poa cf. leioclada)	**33**
33 (32)	Leaf blades folded, apex prominently naviculate (prow-tipped); plants weakly rhizomatous; lemma keels and marginal veins smooth or scaberulous, glabrous; web only	***Poa huancavelicae***
–	Leaf blades flat, apex not, or not prominently, naviculate; plants tufted, occasionally stooling and rooting at nodes (or with prominent sub-rhizomatous shoots present at the base in *Poa leioclada*); lemma keels short pubescent in the lower ½, sometimes sparingly so on the marginal veins near the base	**34**
34 (33)	Spikelets glomerate on branches; culm leaf ligules 1–2 (–2.5) mm long, truncate or obtuse; short sub-rhizomatous shoots usually prominent at the base of the plant, these extravaginal, with brown cataphylls; spikelet proximal lemmas pubescent on keel, lateral, and marginal veins; distal lemmas often pubescent between the veins; spikelets 2–4-flowered; sheaths smooth; leaf blades mostly folded, sometimes flat, firm; lowest floret of spikelets hermaphroditic, upper florets commonly pistillate	**Poa cf. leioclada**
–	Spikelets diffuse throughout the panicle; culm leaf ligules 4–10 mm long, acute, rarely truncate in lower leaves; rhizomatous shoots absent, new shoots obscure, stoloniferous, extravaginal, with green cataphylls; spikelet proximal lemmas glabrous or sparingly pubescent on the keel, and sometimes marginal veins; all lemmas glabrous between the keel and marginal veins; spikelets 2–3-flowered; sheaths nearly smooth to densely scabrous; leaf blades mostly flat, flaccid; all florets of spikelets hermaphroditic	***Poa trivialis***

### Checklist to the open-panicled *Poa* species of Peru

All of the indigenous species covered (including the new ones) are accommodated in Poa subg. Poa supersect. Homalopoa sect. Homalopoa Dumort. s.l., except *Poa calycina* and *Poa swallenii*, which currently reside in Poa subg. Poa supersect. Homalopoa sect. Dissanthelium (Trin.) Refulio, and *Poa hitchcockiana* and *Poa sanchez-vegae*, which reside in Poa subgen. Poa supersect. Homalopoa sect. Dioicopoa subsect. Aphanelytrum. The introduced species are referred to Poa subg. Ochlopoa (Asch. & Graebn.) Hyl. sect. *Micrantherae* Stapf (*Poa annua* and *Poa infirma*); Poa subg. Stenopoa (Dumort.) Soreng & L.J. Gillespie sect. *Pandemos* Asch. & Graebn. (*Poa trivialis*); Poa subg. Poa supersect. Poa sect. Poa (*Poa pratensis*); Poa subg. Stenopoa sect. Tichopoa Asch. & Graebn. (*Poa compressa*).

***Poa aequatoriensis*** Hack. **Ref**: Standley (1936: 125); Tovar (1965: 45, 1993: 135). **Ill**: Hjorth (1991: fig. 6), Tovar (1965: lam. X, B). **Habitat**: Montane forest, Puna grassland, pathsides and open areas, rocky slopes. 2000–4600 m. **Vouchers**: PERU: AMAZONAS: Bongara, *J.J. Wurdack 944* (US). AYACHUCO: *Weberbauer 7573* (US fragm. Ex F). CAJAMARCA: Cajamarca, *I. Sanchez Vega* (MO); Celendin, *I.M. Sánchez V. 2668* (MO); San Miguel, *J. Mostacero L. 1282* (MO); Santa Cruz, *J. Santisteban C. & J. Guevara B. 169* (F, MO). CUSCO: Urubamba, *H.H.C. Ellenberg 48232* (MO). HUÁNUCO: Pachitea, *J.F. Macbride 4365* (US). JUNÍN: Concepción, *J.F. Macbride 3363* (US). PIURA: Huancabamba, *P.M. Peterson 15175* (US). **Discussion**: *Poa aequatoriensis* occurs from northern Peru, Ecuador, and Colombia, although one collection is known from Cusco. Brako and Zarucchi (1993) and Tovar (1993) also state *Poa aequatoriensis* to occur in ANCASH, HUANCAVELICA and LIMA. Commonly misidentified as *Poa trivialis* and vice-versa. Reports of *Poa aequatoriensis* from Bolivia by Hjorth (1991) are, most likely, another taxon. This report was probably based on material called *Poa umbrosa* Trin. by Renvoize (1998; *Renvoize & Cope 4071*, K, US!), which RJS redetermined as *Poa bradei* Pilger, a species otherwise known only from Brazil, which has spikelets with only perfect flowers and short anthers (0.5–1 mm).

***Poa annua*** L. **Syn**: *Ochlopoa annua* (L.) H. Scholz. **Ref**: Standley (1936: 125); Tovar (1965: 61, 1993: 127). **Ill**: Giussani et al. (2012: sp. 3, p. 294). **Habitat**: Waste and disturbed ground, pathsides, roadsides and fields. 2200–4800 m. **Vouchers**: PERU: ANCASH: Carhuaz, *D.N. Smith 9561* (MO); Huari, *P.M. Peterson 13872* (MO, US); Huaylas, *D.N. Smith 9294* (MO); Pallasca, *P.M. Peterson 13947* (MO, US); Recuay, *P.M. Peterson 13827* (MO, US). AREQUIPA: Arequipa, *P.M. Peterson 18256* (US); Caraveli, *P.M. Peterson 16391* (US). AYACUCHO: Huanca Sancos, *P.M. Peterson 16258* (US); Lucanas, *P.M. Peterson 16309* (US); Parinacochas, *P.M. Peterson 16328* (US). CAJAMARCA: Cajamarca, *P.M. Peterson 14910* (MO, US); San Ignacio, *P.M. Peterson 15129* (MO, US). CUSCO: Calca, *S.P. Sylvester 1334* (CUZ, LPB, US, Z); Canchis, *J. Farfán 864* (MO); Cusco, *P. Núñez V. 7500* (US); Espinar, *P. Núñez V. 7619* (MO); La Convención, *S.P. Sylvester 1552* (CUZ, US, Z). HUANCAVELICA: Huancavelica, *P.M. Peterson 14173* (MO, US). JUNÍN: Junín, *P.M. Peterson 14095* (MO, US); Tarma, *D.N. Smith 1605* (MO). LA LIBERTAD: Bolivar, *R.W. Bussmann 18047* (MO); Otuzco, *A. Sagástegui A. 11567* (MO); Trujillo, *J. Hudson 1027* (MO). PASCO: Oxapampa, *D.N. Smith 5831* (MO). PUNO: Chucuito, *P.M. Peterson 14634* (US); El Collao, *P.M. Peterson 14593* (MO, US); Sandia, *B.C. Bennett 1944* (MO). TACNA: Tarata, *P.M. Peterson 14724* (MO, US). **Discussion**: Introduced weed from Europe. This species sometimes survives for more than one growing season and can occur with completely glabrous lemmas, where it is commonly misidentified as *Poa supina* (see ‘Species excluded’ section below). Brako and Zarucchi (1993) and Tovar (1993) also state *Poa annua* to occur in HUÁNUCO, LIMA and PIURA.

***Poa auriculata*** Soreng & P.M. Peterson **Ref & Ill**: Peterson and Soreng (2016: 121, fig. 5). **Habitat**: Known only from the type locality growing on a dry cliff face between 3100–3200 m. **Vouchers**: PERU: AMAZONAS: Chachapoyas, *J.J. Wurdack 1145* (US-holotype). **Discussion**: Endemic herb. This is the first member of Poa subg. Poa supersect. Homalopoa with prominent auricles.

***Poa ayacuchensis*** Tovar **Ref & Ill**: Tovar (1974: 6, 1993: 129, fig. 1, 2, 3). **Habitat**: Wet ground, riversides. c. 3700 m. **Vouchers**: PERU: AYACUCHO: Lucanas, *Ó. Tovar S. & R. Foguel 7007* (MO, US, USM). **Discussion**: Endemic herb, known only from the type collection.

***Poa calycina*** (J. Presl) Kunth **Syn**: *Brizopyrum calycinum* J. Presl; *Deschampsia mathewsii* Ball; *Dissanthelium calycinum* (J. Presl) Hitchc.; *Dissanthelium laxifolium* Swallen & Tovar; *Dissanthelium mathewsii* (Ball) R.C. Foster & L.B. Sm.; *Dissanthelium sclerochloides* Steud. ex E. Fourn.; *Dissanthelium semitectum* Swallen & Tovar; *Dissanthelium supinum* Trin.. **Ref**: Refulio-Rodríguez et al. (2012: 130); Swallen & Tovar (1965: 370–371); Tovar (1993: 154–156). **Ill**: Renvoize (1998: fig. 38 E, F). **Habitat**: Puna grassland. 2500–4500 m. **Vouchers**: PERU: ANCASH: Bolognesi, *P.M. Peterson 17974* (US); Recuay, *P.M. Peterson 17904* (US); Yungay, *P.M. Peterson 21676a* (US). AYACUCHO: Cangallo, *P.M. Peterson 18164* (US); Lucanas, *P.M. Peterson 16466* (US). CUSCO: Calca, *P.M. Peterson 18100* (US); Urubamba, *S.P. Sylvester 1706* (AAU, LPB, US). HUANCAVELICA: Huancavelica, *P.M. Peterson 18100* (US). HUÁNUCO: Huamalies, *P.M. Peterson 17923* (US). JUNÍN: Huancayo, *P.M. Peterson 14209* (US); Junín, *P.M. Peterson 14105* (US); Tarma, *P.M. Peterson 14090* (US); Yauli, *P.M. Peterson 18042* (US). LIMA: Canta, *P.M. Peterson 18028* (US). PASCO: Pasco, *P.M. Peterson 18075* (US). **Discussion**: This species has a disjunct distribution. It is found in the high elevation Puna grasslands of Bolivia and Peru and the alpine volcanic slopes of Mexico (Soreng and Peterson 2012). Brako and Zarucchi (1993) report the species to occur in PUNO.

***Poa candamoana*** Pilg. (Nom. Cons. Prop. *In prep.*) **Syn**: *Poa adusta* J. Presl, lectotype here designated *Haenke s.n.* (lectotype: PR-495759!, left-hand specimen; isolectotype: HAL-81886, W-0029634); *Poa pilgeri* Negritto & Antón. **Ref**: Standley (1936: 125); Tovar (1965: 57, 1993: 131); Negritto and Antón (2006: 88). **Ill**: Tovar (1965: lam. XIII, A); Negritto and Antón (2006: fig. 3, as *Poa pilgeri*). **Habitat**: Grassland. 3400–4500 m. **Vouchers**: PERU: ANCASH: Pacllon, *E. Cerrate 2685* (US); Recuay, *P.M. Peterson 21547* (US); Yungay, *P.M. Peterson 21748* (US). AREQUIPA: Arequipa, *P.M. Peterson 18254* (US). AYACUCHO: Lucanas, *H.H.C. Ellenberg 4945* (MO). CAJAMARCA: Celendin, *P.M. Peterson 21915* (US). CUSCO: Cusco, *A.S. Hitchcock 22469* (US); Espinar, *C. Vargas C. 5629* (MO); Quispicanchis, *P.M. Peterson 20599* (US). HUANCAVELICA: Huancavelica, *P.M. Peterson 16422* (US); Churcampa, *O. Tovar S. 805* (US); Huaytara, *P.M. Peterson 20428a* (US); Tayacaja, *O. Tovar S. 2475* (US). HUÁNUCO: Huamalies, *P.M. Peterson 17925* (US). JUNÍN: Junín, *P.M. Peterson 14116* (US); Huancayo, *I.J. Blair 424* (K), *S. Soukup 6103* (US); Yauli, *Hirsch P243* (US). LIMA: Canta, *P.M. Peterson 20286* (US). MOQUEGUA: Gral. Sanchez Cerro, *D.B. Montesinos T. 2494* (MO, USM). PASCO: Pasco, *P.M. Peterson 14099* (US). PUNO: Azángaro, *A. Weberbauer 472* (MO, US); Puno, *O.P. Pearson 52-68* (US). **Discussion**: A common and characteristic element in the high Andean Puna grassland of Peru and Bolivia. Brako and Zarucchi (1993) state *Poa candamoana* also occurs in APURIMAC.

There has long been uncertainty regarding the identity of *Poa adusta* (Standley 1936, p. 129; Tovar 1965, p. 61, Soreng et al. 2003), which is known only from the type collection, *T. Haenke s.n.* (PR, HAL, W). A report from Colombia (Giraldo-Cañas 2011) represents a different species. The type description of *Poa adusta* can be considered erroneous regarding the lemma indumentum, which was stated to be scabrous while the type specimens examined had pubescence present on the lemma keel marginal and intermediate veins (and at least sparingly between them). After studying the HAL isotype, a solitary flowering shoot with one full leaf, without base, we felt it most likely represented *Poa candamoana*. A photo of the W isotype (which has only an inflorescence and a bit of upper culm) is also a match for the HAL specimen. Upon studying the PR isotype sheet of *Poa adusta*, it was found to comprise two separate leafy shoots that differ from each other. The PR left-hand plant, has a base (with basal-most sheaths characteristic of *Poa candamoana*; ie, the base slightly inflated and lustrous), and it otherwise matches the HAL sample in details. The spikelets of these plants differ from *Poa candamoana* in the broad, blunt, somewhat distorted lemmas, and dark coloration (*adustus* means blackened or scorched), and do not match any species we know of. Although the dark coloration might derive from poor preservation (coming as they did from moldy bundles), we have seen similarly distorted and discolored spikelets in a few specimens of *Poa* from the region that we expect resulted from disease (given that in one case other spikelets in the same plant inflorescence were normal in shape, color, and pubescence). The PR right-hand plant, although bearing similar characteristics of lemma indumentum, is obviously quite pubescent between the veins and the lemmas are acute and not so discolored (a few spikelets are discolored and distorted to a lesser degree than in the left hand plant), lacks a base, has blade apices that are not navicular as is normal for *Poa candamoana*, and the upper culm blade is far too long (longer than the panicle) and inserted too high on the culm for that species (> 10 cm and exceeding the panicle). The right hand plant is almost certainly *Poa horridula*. The left hand plant could be either the result of hybridization between *Poa candamoana* and *Poa horridula* (see *Poa horridula* discussion below), diseased material of *Poa candamoana* or another species, or a rare species not yet rediscovered. Our choice is to lectotypify *Poa adusta* on the left hand plant of specimen PR-495759. We will propose conservation of *Poa candamoana* (Pilger 1906) over *Poa adusta* (J. Presl 1830), rather than outright rejection, since the former name has been widely used for this commonly collected species, and the identity of the lectotype of the latter is still in doubt.

The origin of the *Poa adusta* specimens has been uncertain as they might not have been collected from Peru, even though J. Presl (1830: 271) mentioned ‘Peruvia’ as place of origin in the protologue. The *Poa adusta* specimen at PR was part of a bundle of specimens which were tagged with the note ‘Aus verfault P.’ (translated as ‘from rotten bundle’). The tag, written by Bohemian botanist K.M. Sternberg (sometime between1821 and 1822), indicates that the specimen came from a damaged bundle of plants, for which the country of origin was not indicated. Haenke’s herbarium originally consisted of specimens without labels and, when the Czech National Museum purchased the bundle from the compatriot handling company of Hiecke, Ziencke & Co. in 1821, the origin was indicated only on the top of every bundle of specimens. This is the reason why Haenke’s handwriting is generally missing from all of his specimens. ‘Peruvia’, mentioned in the protologue, is J. Presl’s opinion about the origin of these specimens rather than the real place of origin for some portion of them. Haenke’s collections (made between 1790 and 1792) may have come from his crossing from Buenos Aires and Rio La Plata to Santiago (where he finally caught up with the Malaspina Expedition), or any (suitable) place along the Pacific Coast of America from Santiago (Chile), Lima (Peru), Acapulco (Mexico), to Monterey (California, then part of Mexico), Nootka Sound (now British Columbia), and Yakutat Bay (now Alaska), where the expedition landed (see Češka 2002). Our determination of the right hand plant at PR as *Poa horridula* provides a location for the *Poa adusta* lectotype as the central Andes, where *Poa horridula* and *Poa candamoana* are common.

Although the set of Haenke’s collections that made their way to Prague were purchased by the Czech National Museum, a substantial part of it ended up in the Prague University herbarium (now the herbarium of Charles University in Prague, PRC). J. Presl’s brother (who wrote up the Gramineae, Cyperaceae, and Taccaceae), C.B. Presl, was custodian of the PR herbarium (where all or most of Haenke’s Poaceae from the expedition now reside), and together, while writing the *Reliquiae Haenkeanae* (C.B. Presl 1825–1835), they offered surplus duplicate specimens for sale to other botanical institutions, which is likely how they arrived at HAL and W (Češka 2002; Otakar Sida [PR], *pers. communication*).

***Poa compressa*** L. **Ref**: Soreng and Peterson (2012: 31). **Ill**: Giussani et al. (2012: sp. 11, p. 300). **Habitat**: Cool-temperate, semi-shaded to open habitats on wet ground. **Vouchers**: PERU: JUNÍN: Corpacancha, *G.A. Sullivan 828* (MO). **Discussion**: This species was possibly introduced to the Neotropics for soil stabilization or as a contaminant in other seed. Originating in Europe, and possibly native, but is introduced and naturalized in North America, and Asia. It is presumed to be introduced in Central and South America. This species usually has a congested inflorescence but infrequently exhibits an open inflorescence.

***Poa fibrifera*** Pilg. **Syn**: *Poa carazensis* Pilg.; *Poa ferreyrae* Tovar; *Poa geniculata* Tovar; *Poa tovarii* Soreng. **Ref**: Standley (1936: 126); Tovar (1965: 37–44, 1984: 8, 1993: 128–130); Soreng (1998: 200). **Ill**: Tovar (1965: lam. X, A.; VIII, B, as *Poa ferreyrae*, IX, B as *Poa carazensis*, 1984: fig. 6, 7, 8, as *Poa geniculata*). **Habitat**: Montane forest, Puna grassland, rocky slopes. 2500–4500 m. **Vouchers**: PERU: ANCASH: Aquia, *E. Cerrate 1577* (US); Bolognesi, *E. Cerrate 2607* (US); Carhuaz, *D.N. Smith 9493* (MO); Huaraz, *D.N. Smith 10940* (MO); Huari, *D.N. Smith 12590* (MO); Huaylas, *D.N. Smith 9774* (MO); Pallasca, *P.M. Peterson 13949* (US); Recuay, *P.M. Peterson 13800* (MO, US); Santa, *A. Weberbauer 3073* (MO, US); Ticllos, *E. Cerrate 2650* (US); Yungay, *P.M. Peterson 21741* (US). AYACUCHO: Lucanas, *P.M. Peterson 18210* (US). CAJAMARCA: Cajamarca, *I. M. Sánchez V. 3534* (MO); Celendin, *I. M. Sánchez V. 3488* (MO); Contumaza, *A. Sagástegui 9647* (MO); Hualgayoc, *P.M. Peterson 14941* (MO, US). HUANCAVELICA: Huaytara, *P.M. Peterson 18160* (US). LA LIBERTAD: Contumaza, *E.S. Anderson 1265* (US); Otuzco, *R. Ferreyra 7619* (MO, US); Santiago de Chuco, *A. Sagástegui A. 11809* (MO). LIMA: Canta, *P.M. Peterson 20262* (US); Huarochiri, *R. Ferreyra 8252* (US). **Discussion**: Endemic herb (a voucher from Bolivia, *T. Johns 82-108*, was redetermined by RJS as *Poa horridula*). *Poa ferreyrae* and *Poa geniculata* (=*Poa tovarii*), known only from the type collections at La Libertad-Otuzco, and *Poa carazensis*, known from the departamento of Ancash, are seen to be indistinct from *Poa fibrifera* and so have been placed as synonyms of this species. Brako and Zarucchi (1993) also state *Poa fibrifera* to occur in JUNÍN and HUÁNUCO. Specimens from JUNÍN originally identified as *Poa fibrifera* (*A.S. Hitchcock 22176*; *P.M. Peterson 14227*) have been redetermined as *Poa glaberrima* and *Poa kurtzii*, respectively. It is impossible to say if either of these specimens were accepted by Brako and Zarucchi (1993) as only one specimen was cited for the country.

***Poa gilgiana*** Pilg. **Syn**: *Melica expansa* Steud. ex Lechl.. **Ref**: Standley (1936: 126); Tovar (1965: 36, 1993: 128). **Ill**: Tovar (1965: lam. VIII, A). **Habitat**: Grassland. 3700–4700 m. **Vouchers**: PERU: AREQUIPA: Arequipa, *P.M. Peterson 18255* (US); Caraveli, *P.M. Peterson 16394* (US). JUNÍN: Junín, *A.S. Hitchcock 22251* (US). LIMA: Canta, *P.M. Peterson 18025* (US). PUNO: Azángaro, *A. Weberbauer 477* (MO, US); Chucuito, *P.M. Peterson 14678* (US). TACNA: Tarata, *P.M. Peterson 14830* (MO, US). **Discussion**: Distributed in high elevation Puna grasslands from central Peru to Bolivia. Possibly a variety of *Poa horridula* which needs further study. This species is suspected to hybridize with *Poa horridula* with intermediate forms being collected from ANCASH-Aquia (*E. Cerrate 1564*, US), HUANCAVELICA-Huaytara (*P.M. Peterson 20424a*, US) and PUNO-Chucuito (*P.M. Peterson 14669*, MO, US).

***Poa glaberrima*** Tovar **Ref**: Tovar (1965: 40, 1993: 129). **Ill**: Tovar (1965: lam. IX, A). **Habitat**: Puna tussock grassland, humid areas, wet ground. 3300–4700 m. **Vouchers**: PERU: ANCASH: Recuay, *P.M. Peterson 13821* (MO, US). APURIMAC: Ayamaraes, *P.M. Peterson 16507* (US). AYACUCHO: Huamanga, *P.M. Peterson 20532a* (US). CUSCO: Calca, *P.M. Peterson 16555* (US). HUANCAVELICA: Huancavelica, *P.M. Peterson 14168* (MO, US). JUNÍN: Huancayo, *Anonymous 34* (US); Junín, *D.N. Smith 5649* (MO); Tarma, *D.N. Smith 2989* (MO); Yauli, *P.M. Peterson 14044* (US). LIMA: Canta, *P.M. Peterson 20282* (US); Huarochiri, *J.R. Swallen 7068* (US). PUNO: Lampa, *P.M. Peterson 20744* (US); Melgar, *P.M. Peterson 20721* (US); Puno, *H.H.C. Ellenberg 249* (US). **Discussion**: Previously considered endemic, but has been found in Ecuador, Bolivia, and Argentina according to Giussani et al. (2012: 304); we have verified specimens from Bolivia (*S.A Renvoize 4475*, US) and Argentina (*P.M. Peterson et al. 19577*), but not from Ecuador. This species superficially resembles *Poa candamoana* but the latter prefers dry ground and has hairy lemmas and lacks extravaginal shoots. Certain specimens placed under this name from southern Peru (eg. PUNO: Lampa “2 km SW of San Jose on road towards Junin” *P.M. Peterson 20744*, [US]; Melgar “ca. 7 km WNW of Santa Rosa on Hwy 3 and 1 km W toward Quishuara, along Rio Santa Rosa” *P.M. Peterson 20624b* [US]), Bolivia and northern Argentina (*P.M. Peterson 19577* [US]; Giussani et al. 2012, Figure pg. 304) have odd forms with sparse inflorescences and may actually be a species new to science. They resemble *Poa pauciflora*, but have completely glabrous and smooth spikelets. Future study should focus on verifying the status of the southern *Poa glaberrima* populations.

***Poa grisebachii*** R.E. Fr. **Syn**: *Poa superata* Hack. **Ref**: Giussani et al. (2012: 305, 336). **Ill**: Negritto and Antón (2000: fig 1.); Giussani et al. (2012: sp. 45, p. 305; sp. 59, p. 366, as *Poa superata*). **Habitat**: Dry Puna grasslands and high Andean steppe. 3000–4500 m. **Vouchers**: PERU: AYACUCHO: Parinacochas, *P.M. Peterson 16323* (US). JUNÍN: Yauli, *Ó. Tovar S. 6447* (MO). MOQUEGUA: Carumas, *A. Weberbauer 7310* (US); Mariscal Nieto, *P.M. Peterson 14553* (US). TACNA: Tarata, *P.M. Peterson 14793* (MO, US). **Discussion**: Type collections were verified of *Poa grisebachii* (ARGENTINA: JUJUY: Santa Catalina, *F. Kurtz 11412*, US fragm. ex S!) and *Poa superata* (ARGENTINA: TUCUMÁN: Tafí, Cumbres Calchaquíes, *T.J.V. Stuckert Herb. ARG. 17738 ex Lillo 5604*, US fragm. ex W!). Other collections were verified from Peru (see vouchers above), Chile (ARICA & PARINACOTA: Zapahuira, *P.M. Peterson 15700*, US; TARAPACÁ: above Pucapa, *P.M. Peterson 15615*, US) and Argentina (JUJUY: Santa Catalina, *F. Kurtz 11409*, US (fragm. ex S); SALTA: Pampa Grande, *C. Spegazzini 60*, US). Previously, *Poa grisebachii* and *Poa superata* were both considered endemic to Argentina (Giussani et al. 2012) and this is the first recording of the species from Peru. It is likely that, with further revision of Bolivian *Poa*, this species will also be found to occur in Bolivia. Specimens of *Poa superata* were found to be morphologically indistinct from the type of *Poa grisebachii* and so have been grouped as a single taxon. All material examined of this taxon exhibited soft pubescence on at least the upper lemmas of the spikelets, with the lower florets being glabrous or softly pubescent. *Poa grisebachii* can be highly variable in terms of lemma pubescence. The lectotype of *Poa grisebachii* p.p. has glabrous and smooth lemmas while the isolectotype and other syntypes of *Poa grisebachii* p.p. at US fragms. ex UPS, and the type specimens of *Poa superata* p.p., were all observed with at least sparsely pubescent lemmas. All the Peruvian material of *Poa grisebachii* has pubescent lemmas, at least on the distal florets.

The plants resemble *Poa fibrifera*, and are sometimes fibrous at their bases, but can usually be distinguished by the distinctly pubescent lemmas. The leaf blades are usually somewhat thin, flat, lax, and scabrous, 1.5–3.5 mm wide, tapering to a very slender apex. Certain specimens were found from AYACUCHO: Lucanas, e.g. *P.M. Peterson 16317* (US) that bore characteristics of *Poa horridula*. Another specimen from MOQUEGUA: Mariscal Nieto, *P.M. Peterson 14553* (US), could also not be confidently placed to the species. This species may also hybridize with *Poa kurtzii*, with intermediate forms being collected which exhibit narrower, firmer, involute blades and generally smaller and more scabrous spikelets.

***Poa hitchcockiana*** Soreng & P.M. Peterson **Syn**: *Brachyelytrum procumbens* Hack.; *Aphanelytrum decumbens* Hack. ex Sodiro. **Ref & Ill**: Peterson and Soreng (2016: 111, fig. 1). **Habitat**: Paramo or moist Jalca vegetation between 2000–4025 m. **Vouchers**: PERU: CUSCO: Paucartambo, *P.M. Peterson 16571* (US, USM); Calca, *P.M. Peterson 16581* (US, USM); Pillco, *C. Vargas C. 19264* (US); Quispicanche, *P.M. Peterson 20582* (US, USM). MOQUEGUA: El Abra, *C. Vargas C. 19104* (US). SAN MARTÍN: Huicungo, *A. Cano s.n.* (SI, USM); *B. León 3797* (USM). **Discussion**: The only wide ranging species of Poa subsect. Aphanelytrum, this species is found from the Andes of Colombia, Ecuador, Peru, and Bolivia. *Poa hitchcockiana* also has the most unusual spikelet morphology with extremely long rachillas (1.5–4.2 mm long) and very short [0.1–0.5 (–0.7) mm long] to obscure or often absent, unveined glumes.

***Poa horridula*** Pilg. **Syn**: *Melica expansa* Steud ex Lechl.; *Poa dumetorum* Hack.; *Poa piifontii* J. Fernandez Casas, J. Molero & A. Susanna; *Poa pufontii* Fern. Casas, Molero & Susanna; *Poa unduavensis* Hack.. **Ref**: Standley (1936: 127); Tovar (1965: 53, 1993: 131). **Ill**: Hjorth (1991: fig. 4), Tovar (1965: lam. XII, B). **Habitat**: Grasslands, rocky slopes, shrublands. 2900–4700 m. **Vouchers**: PERU: ANCASH: Bolognesi, *P.M. Peterson 17888* (US); Pallasca, *P.M. Peterson 21810* (US); Recuay, *P.M. Peterson 13841* (MO, US); Santa, *A. Weberbauer 3113* (MO, US); Yungay, *P.M. Peterson 21631* (US). APURIMAC: Abancay, *P.M. Peterson 16645* (US); Aymaraes, *P.M. Peterson 16477* (US). AREQUIPA: Caylloma, *P.M. Peterson 18298* (US). AYACUCHO: Huamanga, *P.M. Peterson 20503* (US); Huanca Sancos, *P.M. Peterson 16283* (US); Lucanas, *P.M. Peterson 16289* (US); Parinacochas, *P.M. Peterson 16346* (US). CAJAMARCA: Cajamarca, *I.M. Sánchez V. 79* (US); Celendin, *I.M. Sánchez V. 2911* (MO); Hualgayoc, *P.M. Peterson 14938* (US). CUSCO: Anta, *G.R. Brunel 283* (MO); Calca, *S.P. Sylvester 1021* (CUZ, US, Z); Cusco, *A.S. Hitchcock 22443* (US); La Convención, *S.P. Sylvester 2028* (LPB, US, Z); Paruro, *P. Núñez V. 7365* (MO); Quispicanchis, *P.M. Peterson 20549* (US); Urubamba, *H.H.C. Ellenberg 4824* (MO). HUANCAVELICA: Huancavelica, *P.M. Peterson 14175* (MO, US); Huaytara, *P.M. Peterson 18158* (US); Tayacaja, *O. Tovar S. 4213* (MO). HUÁNUCO: Huamalies, *P.M. Peterson 17922* (US); Yarowilca, *J.F. Macbride 1970* (US). JUNÍN: Huancayo, *Black 46-711* (US); Jauja, *P.M. Peterson 14122* (US); Junín, *A.S. Hitchcock 22213* (US). LA LIBERTAD: Bolivar, *P.M. Peterson 21944* (US). LIMA: Canta, *P.M. Peterson 20278* (US); Huarochiri, *P.M. Peterson 14018* (MO, US); Yauyos, *E. Cerrate 1083* (US). PASCO: Daniel Alcides Carrión, *A.S. Hitchcock 22302* (US); Pasco, *A.S. Hitchcock 22260bis* (US). PUNO: Chucuito, *P.M. Peterson 14672* (US); Puno, *H.H.C. Ellenberg 342* (US); Sandia, *B.C. Bennett 2323* (MO). **Discussion**: Found above 3000 m in Puna and Paramo grasslands from Colombia to Bolivia. Brako and Zarucchi (1993) and Tovar (1993) indicate that *Poa horridula* also occurs in MOQUEGUA. This species is suspected to hybridize with *Poa gilgiana* (see *Poa gilgiana* discussion, above) and *Poa candamoana*, due to the presence of intermediate forms across the ranges of these species. Specimens *Hirsch P1399* (US) and *E. Cerrate 2285* (US) appear to be intermediates between *Poa horridula* and *Poa candamoana* and could be either a short *Poa horridula* or a robust *Poa candamoana*.

***Poa huancavelicae*** Tovar **Syn**: *Poa paramoensis* Lægaard. **Ref**: Tovar (1965: 52, 1993: 134); Lægaard (1998: 28). **Ill**: Tovar (1965: lam. XII, A); Lægaard (1998: fig. 2, as *Poa paramoensis*). **Habitat**: Puna grassland and *Polylepis* woodland. 4000–4800 m. **Vouchers**: PERU: ANCASH: Bolognesi, *P.M. Peterson 17953* (US); Huaraz, *D.N. Smith 10839* (MO); Recuay, *P.M. Peterson 21540* (US); Yungay, *P.M. Peterson 21766* (US). CUSCO: Calca, *P.M. Peterson 16604* (US); Carhuaz, *D.N. Smith 11206* (MO); La Convención, *S.P. Sylvester 1565* (US, Z); Quispicanchis, *P.M. Peterson 20589* (US); Urubamba, *H.H.C. Ellenberg 449* (MO). HUANCAVELICA: Castrovirreyna, *Ó. Tovar S. 28* (MO). **Discussion**: Previously considered endemic to Peru and found in HUANCAVELICA and CUSCO (Brako and Zarucchi 1993; Tovar 1993). *Poa paramoensis* was found to be inconsistently morphologically distinct from *Poa huancavelicae*; the northern plants branches are usually fairly smooth, but the variation appears to be continuous to more scabrous forms further south in Peru, and so it has been synonymized. Specimens from La Convención, CUSCO, have proximal lemmas much shorter (3–3.5 mm) than in the original species description (4–4.5 mm) and anthers were also much shorter (1–1.3 mm). These plants were also found to be short-rhizomatous/sub-rhizomatous, while *Poa paramoensis* p.p. is tufted and the isotype material of *Poa huancavelicae* p.p. at MO appears tufted, although Tovar (1993) mentions it to be rhizomatous.

***Poa infirma*** Kunth **Syn**: *Catabrosa thomsonii* Hook. f.; *Colpodium thomsonii* (Hook. f.) Hack.; *Eragrostis infirma* (Kunth) Steud.; *Megastachya infirma* (Kunth) Roem. & Schult.; *Ochlopoa infirma* (Kunth) H. Scholz; Poa annua subsp. exilis (Tomm. ex Freyn) Asch. & Graebn.; *Poa exilis* (Tomm. ex Freyn) Murb.; *Poa remotiflora* (Hack.) Murb.. **Ref**: Müller et al. (1981: 334); Tovar (1986: 56; 1993: 126). **Ill**: Giussani et al. (2012: sp. 27, p. 311). **Habitat**: Dry forest, Puna grassland. c.4400 m. **Vouchers**: PERU: CUSCO: Calca, *P. Núñez V. 7063* (MO), *S.P. Sylvester 1390* (US, Z). **Discussion**: Introduced from Europe. Brako and Zarucchi (1993) and Tovar (1993) state *Poa infirma* occurs in HUANCAVELICA and LIMA, with the specimens from LIMA being found on coastal hills in sandy soil. *Poa infirma* is the maternal diploid parent of *Poa annua* (tetraploid), with *Poa supina*, another diploid, being the plastid donor (Soreng et al. 2010).

***Poa kurtzii*** R.E. Fr. **Syn**: *Poa altoperuana* R. Lara & Fern. Casas; *Poa asperiflora* Hack.; *Poa munozensis* Hack.; *Poa pflanzii* Pilg. **Ref**: Standley (1936: 125); Tovar (1965: 60, 1993: 133); Giussani et al. (2012: 312). **Ill**: Negritto and Antón (1999: fig. 2); Giussani et al. (2012: sp. 30, p. 313); Tovar (1965: lam. XII, A, as *Poa asperiflora*). **Habitat**: High Andean Puna grassland, rocky slopes. 3300–5100 m. **Vouchers**: PERU: ANCASH: Huari, *D.N. Smith 10138* (MO); Recuay, *P.M. Peterson 21510* (US). AREQUIPA: Arequipa, *P.M. Peterson 18260* (US); Caraveli, *P.M. Peterson 16393* (US). AYACUCHO: Huamanga, *P.M. Peterson 20531* (US); Huanca Sancos, *P.M. Peterson 16244* (US); Lucanas, *P.M. Peterson 16177* (US); Parinacochas, *P.M. Peterson 16345* (US). HUANCAVELICA: Castrovirreina, *O. Tovar S. 2838* (US); Huancavelica, *P.M. Peterson 16416* (US); Huaytara, *P.M. Peterson 20426* (US). JUNÍN: Huancayo, *P.M. Peterson 14227* (US). LA LIBERTAD: Trujillo, *H.H.C. Ellenberg 3779* (MO). MOQUEGUA: Mariscal Nieto, *P.M. Peterson 14552* (US), *P.M. Peterson 18312* (US). PUNO: Azángaro, *H.H.C. Ellenberg 598* (US); Chucuito, *P.M. Peterson 14677* (US); El Collao, *P.M. Peterson 14591* (US). TACNA: Tacna, *P.M. Peterson 14762* (US); Tarata, *P.M. Peterson 14727* (US). **Discussion**: This species is found from Peru to Argentina. JUNÍN specimen *P.M. Peterson 14227* is atypical and requires further study. Brako and Zarucchi (1993) state *Poa asperifolia* (=*kurtzii*) to also occur in CUSCO and LIMA. The species is highly variable in terms of lemma indumentum, and is found in two leads in both keys (see Appendix 1) to account for this.

***Poa*** cf. ***leioclada*** Hack. **Ref**: Hitchcock (1927). **Ill**: Hjorth (1991: fig. 8) **Habitat**: Humid Paramo grasslands. 3200–4300 m. **Vouchers**: PERU: ANCASH: Yungay, *D.N. Smith 9095* (MO); Huaylas, *D.N. Smith 9320a* (MO). PIURA: Huancabamba, *P.M. Peterson 15175* (US). **Discussion**: Specimens are an imperfect match of Ecuadorian material of *Poa leioclada* as they bear characteristics of both *Poa mulalensis* Kunth and *Poa leioclada*. Both these species were previously considered Ecuadorian endemics. This taxon needs further study.

***Poa oscariana*** Negritto & Antón **Ref & Ill**: Negritto and Antón (2006: 84 [88], fig. 2). **Habitat**: Puna grassland, rocky slopes. 3600–4100 m. **Discussion**: Endemic herb, only known from CUSCO, Paucartambo. None of the type or paratype specimens have been examined. This may be a variety of *Poa gilgiana*, but further study is needed.

***Poa pauciflora*** Roem. & Schult. **Syn**: *Poa depauperata* Kunth; *Poa pardoana* Pilg. **Ref**: Standley (1936: 128); Tovar (1965: 49, 1993: 133). **Ill**: Hjorth (1991: fig. 11), Tovar (1965: lam. XI, B, as *Poa pardoana*). **Habitat**: Puna grassland, rocky slopes. 3200–4900 m. **Vouchers**: PERU: ANCASH: Corongo, *P.M. Peterson 21777* (US); Huaraz, *D.N. Smith 10797* (MO); Huari, *P.M. Peterson 13885* (MO, US); Huaylas, *D.N. Smith 9930* (MO); Pallasca, *P.M. Peterson 21842* (US); Recuay, *P.M. Peterson 13848* (MO, US); Yungay, *P.M. Peterson 21678* (US). CAJAMARCA: Cajamarca, *P.M. Peterson 14887* (US), *H.H.C. Ellenberg 1825* (US); Celendin, *P.M. Peterson 21905* (US); San Miguel, *P.M. Peterson 14933* (US); San Pablo, *P.M. Peterson 14878* (US). JUNÍN: Tarma, *P.M. Peterson 14065* (US). LA LIBERTAD: Bolivar, *P.M. Peterson 21936* (US); Sanchez Carrion, *D.N. Smith 2242* (MO); Santiago de Chuco, *P.M. Peterson 13953* (US). SAN MARTÍN: Mariscal Caceres, *B. León 1654* (MO). **Discussion**: Specimens have also been verified from Ecuador, but it is unclear whether this species extends to Colombia and Bolivia.

***Poa pearsonii*** Reeder **Ref**: Tovar (1965: 33, 1993: 127); Giussani et al. (2012: 325). **Ill**: Giussani et al. (2012: sp. 45, p. 325). **Habitat**: Rocky Puna grassland. 4500–4900 m. **Vouchers**: PERU: PUNO: El Collao, *O.P. Pearson 91* (US). TACNA: Tacna, *P.M. Peterson 13953* (MO, US). **Discussion**: Found from southern Peru, Bolivia, Argentina, and is here reported for Chile (*P.M. Peterson 15676*). Brako and Zarucchi (1993) state *Poa pearsonii* to also occur in LIMA and AREQUIPA.

***Poa pratensis*** L. **Ref**: Standley (1936: 128); Tovar (1993: 135); Giussani et al. (2012: 328). **Ill**: Giussani et al. (2012: sp. 50, p. 329). **Habitat**: Open Andean grasslands. 3500–3900 m. **Vouchers**: PERU: CAJAMARCA: Cajamarca, *I.M. Sánchez V. 2668* (MO); San Miguel, *P.M. Peterson 14921* (MO, US). HUANCAVELICA: Huancavelica, *P.M. Peterson 18107* (US). JUNÍN: Huancayo, *P.M. Peterson 14223* (US); Yauli, *D.N. Smith 2979* (MO). **Discussion**: Plants in Peru are presumably introduced from Europe, but the species in tropical latitudes tends to look odd and cannot be confidently placed to subspecies. Poa pratensis subsp. alpigena (Lindm.) Hiitonen is apparently native in North and South America (in Patagonia). Brako and Zarucchi (1993) state *Poa pratensis* to also occur in PUNO.

***Poa ramifera*** Soreng & P.M. Peterson **Ref. & Ill**: Soreng and Peterson (2010: 587, fig. 1, 2, 3). **Habitat**: Shrublands. 2700–3100 m. **Vouchers**: PERU: ANCASH: Corongo, *P.M. Peterson 21804* (MO, US, USM). **Discussion**: Endemic, known only from the type locality.

***Poa sanchez-vegae*** Soreng & P.M. Peterson **Syn**: *Aphanelytrum peruvianum* Sánchez Vega, P.M. Peterson, Soreng & Lægaard. **Ref & Ill**: Peterson and Soreng (2016: 118, fig. 4). **Habitat**: Rocky sites associated with Jalca vegetation (humid alpine grass ecosystems) at 3300 m. Vouchers: PERU: CAJAMARCA: Cajamarca, *I. Sánchez-Vega 11781* (CPUN, AAU, F, HAO, HUT, LOJA, MICH, MO, SI, US, USM). **Discussion**: Endemic, known only from the type locality.

***Poa scabrivaginata*** Tovar **Ref**: Tovar (1965: 48, 1993: 134). **Ill**: Tovar (1965: lam. XI, A). **Habitat**: Shrublands. c. 4000 m. **Vouchers**: PERU: HUÁNUCO: Pachitea, Tambo de Vaca, *J.F. Macbride 4354* (MO, US-holotype). **Discussion**: Endemic herb. Known only from the type collection. A paratype from HUÁNUCO, and further specimens sometimes referred to as *Poa scabrivaginata* from CAJAMARCA (e.g. Celendin, *I.M. Sánchez V. 2668* [MO]; Santa Cruz, *J. Santistaban C. & J. Guevara B. 169* [MO]) and JUNÍN (e.g. Concepción, *J.F. Macbride 3363* [US]) are better included in *Poa aequatoriensis*. In the *Poa scabrivaginata* type, lower sheaths are densely and coarsely scabrous, glumes are long (lower 3.5–4 mm, upper 4–4.5 mm), lemmas are glabrous except for the web and smooth except for sparsely scaberulous upper keel and apex, and anthers are 1.5–2 mm long. The other material has shorter glumes, sheaths smooth or lightly scabrous (often scabrous only on the margins), lemmas usually with pubescence on the keel and marginal veins, and anthers usually shorter.

***Poa swallenii*** Refulio **Syn**: *Dissanthelium expansum* Swallen & Tovar. **Ref**: Refulio- Rodríguez et al. (2012: 130); Swallen and Tovar (1965: 374); Tovar 1993: 157). **Habitat**: Puna grasslands. 3600-4600 m. **Vouchers**: PERU: CUSCO: Calca, *P.M. Peterson 16594* (US), *P.M. Peterson 16569* (US), *P.M. Peterson 16612* (US); La Convención, *O.F. Cook 1305* (US), *S.P. Sylvester 1924* (US, USM, Z); Urubamba, *S.P. Sylvester 1071* (CUZ, US, Z). **Discussion**: Endemic herb. Swallen and Tovar (1965) and Tovar (1993) cite a collection of *Macbride & Featherstone 2183* from HUÁNUCO, and *P.C. Hutchison 1215* from LIMA.

***Poa trivialis*** L. subsp. ***trivialis* Ref**: Standley (1936: 129); Tovar (1993: 134); Giussani et al. (2012: 336). **Ill**: Giussani et al. (2012: sp. 60, p. 337). **Habitat**: Andean slopes at middle elevations. **Vouchers**: PERU: JUNÍN: *A.S. Hitchcock 22277* (US), *A.S. Hitchcock 22279* (US), *A.S. Hitchcock 22266a* (US). **Discussion**: Introduced from Europe. Brako and Zarucchi (1993) state *Poa trivialis* to also occur in CAJAMARCA. However, the collection cited by Brako and Zarucchi (1993), *J. Mostacero L. 1282* (MO), was redetermined as *Poa aequatoriensis*. *Poa trivialis* and *Poa aequatoriensis* bear many superficial resemblances and can be easily confused. The Hitchcock specimens had no habitat type or specific locality mentioned.

***Poa trollii*** (Pilg.) Refulio **Syn**: *Dissanthelium trollii* Pilg. **Ref**: Tovar (1986: 51; 1993: 153). **Ill**: Renvoize (1998: fig. 38 G, H). **Habitat**: Dry Puna grassland. 4400–4800 m. **Vouchers**: PERU: PUNO: El Collao, *P.M. Peterson 18303* (US). **Discussion**: Distributed in the high Andean dry Puna grassland in southern Peru and the Potosi, Bolivia. This species normally has congested, spikelike panicles but has been collected with open panicles during anthesis.

### Newly described species

#### Poa ramoniana

Taxon classificationPlantaePoalesPoaceae

Soreng & S.P. Sylvester sp. nov.

urn:lsid:ipni.org:names:77155738-1

Fig. 1

##### Type.

PERU. Región JUNÍN. Prov. Junín: Distr. Carhuamayo, orillas de la laguna de Capillacocha, {est. vicinity: S10.86443°, W75.99256°} entre Carhuamayo y Paucartambo, Puna grassland, 4200–4300 m, 8 Jan 1949, *R. Ferreyra 5200 p.p. a* (holotype: US-2207173!; isotype: USM p.p.)

Plants gynomonoeious. **Perennials**; Rhizomatous, with well developed, slender, lateral tending, rhizomes, small tufted. **Tillers** extravaginal. **Culms** c. 5 cm tall, erect, unbranched, isolated or two together; Culm nodes terete, smooth, included in the sheaths; Culm internodes less than 1 cm long (peduncle c. 3 cm), terete, smooth. **Leaves** equally basal and cauline; Sheaths slightly laterally compressed, keeled, smooth, glabrous; Butt sheaths papery or slightly fibrous in age; Uppermost culm sheath c. 18 mm long, margins fused c. 40% their length, distal sheaths longer than their blades; Collars and throats smooth, glabrous; Ligules 1–1.5 mm long, sometimes with a central dent to 2 mm long, indistinctly decurrent, abaxially moderately densely scabrous, apices obtuse, margin irregular sometimes with a tooth, of sterile shoots similar to those of the culm; Cauline blades to 2 cm long, mostly folded or infrequently flat, with strongly inrolled margins, abaxially, marginally, and adaxially smooth, glabrous, tips distinctly prow shaped; Blades graduated up the culm, the sub-terminal one the longest; Sterile shoot blades to 4.5 cm long. **Panicles** 2–2.5 cm long, open, exerted, c. 1 cm wide, with 13–15 spikelets, proximal internode c. 6 mm long, weakly scabrous angled; Rachis with 1 branch per node; Primary branches spreading to reflexed, the upper ones ascending, distinctly angled, closely scabrous along the angles; Lateral pedicels less than 0.5 mm long, scabrous angled; Longest branches c. 7 mm, with 4–5 spikelets clustered in the distal half. **Spikelets** 3.5–4 mm long, c. 1.5 × longer than wide, broadly ovate, laterally compressed, not bulbiferous, anthocyanic and bronzy; Florets (2–)3, the proximal 1 (or 2 if 3 total) perfect, the distal 1 pistillate; Rachilla internodes 0.4–0.8 mm long, terete, distal internodes terete, smooth, glabrous, mostly hidden; Glumes more or less equal, both broadly lanceolate, or the first lanceolate, sub-lustrous in the scareous-hyaline margins, distinctly keeled, keels smooth or distally smooth or sparsely scaberulous, apices acute and pointed to obtuse and blunt and denticulate; Lower glumes 2.5–3 mm long, 1–3-veined; Upper glumes 3–3.2 mm long, subequal in width to the lower, 2–3-veined; Calluses glabrous; Lemmas (the lowest) 2.9–3.5, 5-veined, broadly lanceolate to ovate, green proximally, anthocyanic distally with a wide bronzy band apically, strongly laterally compressed, distinctly keeled, thin, keel smooth or obscurely scaberulous distally, smooth or mostly smooth elsewhere, glabrous, intermediate veins distinct, not extending into the scarious apical margin, edges smooth, apices scarious-hyaline bronzy for the distal c. 1 mm, edges smooth or slightly erose to denticulate, obtuse, blunt or slightly pointed; Paleas to 1 mm shorter than the lemma, glabrous, keels smooth or distally sparsely scaberulous. **Flowers** chasmogamous; Lodicules not observed; Anthers c. 2 mm long (vestigial in pistillate flowers). **Caryopses** unknown. 2*n* = unknown.

##### Distribution.

Known only from a single locality in Junín.

##### Habitat.

Puna grassland, 4200–4300 m, in wet margins/shore of lakes, in moss.

##### Etymology.

The species is named in recognition of the eminent Peruvian botanist, Ramón Alejandro Ferreyra (1910–2005) who collected the type and paratype.

##### Conservation status.

Data insufficient.

##### Additional specimens examined.

PERU. Región JUNÍN. Prov. Junín: Distr. Carhuamayo, Capillacocha cerca a Carhuamayo, Puna grassland, 4200–4300 m, 8 Jan 1949, *R. Ferreyra 5211* (USM!).

##### Discussion.

This new species appears like a small form of *Poa glaberrima*, but differs by being extensively rhizomatous and reaching only 5 cm tall, versus densely tufted and 12–45 cm tall for *Poa glaberrima*. The US holotype of *Poa ramoniana* is a mixed collection, with a second taxon p.p. “b”, which appears to be *Poa gymnantha* Pilg. that is sterile, tightly tufted, with intravaginally branching shoots and involute leaf blades that are adaxially scabrous. The USM isotype also contains two species: the small rhizomatous plant is *Poa ramoniana*; the taller plants appear to be *Poa glaberrima*. Tovar originally determined the USM type and paratype as *Poa lilloi* (Tovar 1993), which, among other differences, has a dense habit, without rhizomes, and ascending panicles branches, densely scabrous lemmas with narrow white, scarious margins, and sometimes a web on the callus. Tovar also identified the US type as *Poa ovata* Tovar (1965: 17), which RJS considers to be a rare to uncommon sexually reproducing phase of the small form of *Poa gymnantha*, a species that is otherwise predominantly pistillate and apomictic (Negritto et al. 2008). Other material determined as *Poa lilloi* in Peru has been referred to *Poa glaberrima* and *Poa candamoana*, or small *Poa kurtzii* (see excluded species, below).

**Figure 1.**
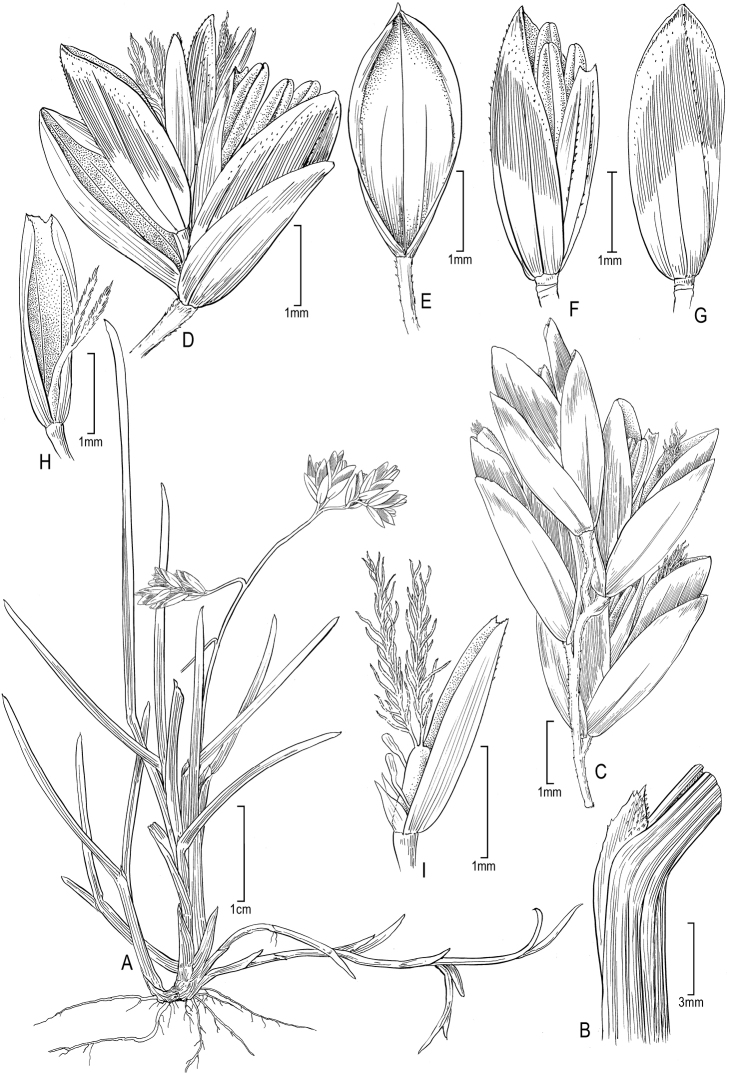
*Poa ramoniana*. **A** Habit **B** Ligular zone **C** Terminal segment of inflorescence branch with four spikelet cluster **D** Spikelet **E** Lower glume ventral view **F** Proximal floret (perfect) **G** Upper glume dorsal view **H** Palea with immature pistil (pistillate floret), dorsal view **I** Palea with pistil (pistillate floret), staminodes, and lodicules, lateral view. Drawn from type material (*R. Ferreyra 5200 p.p. a*, US-2207173).

#### Poa tayacajaensis

Taxon classificationPlantaePoalesPoaceae

Soreng & S.P. Sylvester sp. nov.

urn:lsid:ipni.org:names:77155739-1

Figs 2 , 3

##### Type.

PERU: Región HUANCAVELICA. Prov. Tayacaja: Distr. Colcabamba, Chuspi-Hda., Tocas, entre Colcabamba y Paucarbamba, monte bajo, 2900 m, [vic. S12.5°, W74.6°], 22 Apr 1954, *Ó. Tovar. S. 2038* (holotype: US-2181284!; isotype: USM p.p.)

Plants gynomonoecious. **Perennials**; tufted, without lateral or downward tending shoots. **Tillers** intravaginal. **Culms** 55–65 cm tall, erect or decumbent (when decumbent sometimes extravaginally branching at the lower culm nodes, i.e. *Peterson et al. 20369*); Culm nodes 3–4, terete, smooth, 2–3 nodes exposed at flowering; Culm internodes terete, smooth. **Leaves**; Sheaths slightly laterally compressed, keeled, lower culm and lateral ones densely scaberulous distally; Butt sheaths thin papery, somewhat loosely investing the shoots; Uppermost culm sheaths 10–13 cm long, margins fused 60–75 % their length, slightly shorter than their blades; Collars and throats smooth, glabrous; Ligules 2.0–3.5 mm long, not decurrent, abaxially sparsely to moderately densely scabrous, apices obtuse to subacute, margins densely scabrous, ligules of sterile shoots and lower culm leaves 0.5–1 mm long; Cauline blades 6–15 cm long, 3–5 mm wide, well developed, longer than their sheaths, generally flat, keeled, thin, lax, abaxially, marginally, and adaxially scabrous mainly along the veins, folded near the apex, apex gradually tapered to a slender point; Blades gradually increasing in length up the culm, flag leaf blade 10–15 cm long; Sterile shoot blades like those of the culm, but somewhat shorter and smoother. **Panicles** 18–20 cm long, loose, open, exerted, slightly lax, to 5 cm wide, with more than 100 spikelets, proximal internodes 3.5–4 cm long, smooth; Rachis with (3) 5–6 branches at lower nodes; Primary branches slender, mostly laxly ascending, sometimes spreading, one sometimes reflexed, angled, proximally smooth to moderately scabrous mainly on the angles; Lateral pedicels mostly < 1 mm long, scabrous; Longest branches 6–8 cm long, with 14–22 spikelets in the distal half, slightly overlapping. **Spikelets** 4.5–6 mm long, c. 2 × longer than wide, lanceolate, laterally compressed, not bulbiferous, greyish-green to somewhat anthocyanic at maturity; Florets (3–)4(–5), proximal florets hermaphroditic and distal one pistillate; Rachilla internodes terete, distal internodes 0.7–1 mm long, terete, smooth, glabrous; Glumes unequal, narrow lanceolate to lanceolate, herbaceous and pale green below, sometimes anthocyanic in margins and apex, veins distinct, distinctly keeled, keels sparsely short scabrous distally, surfaces smooth, margins scarious-hyaline, edges entire smooth, apices sharply acute, entire; Lower glumes 1.7–2.5 mm, 2/3–4/5 as long as adjacent lemmas, 1-veined, very narrow, slightly sickle shaped; Upper glume 2.4–3 mm, c. 2 × wider than the lower, 3-veined; Calluses webbed, with a dense, long dorsal tuft of wooly hairs; Lemmas (the lowest) 2.8–3.7 mm long, 5-veined, lanceolate in side view, the proximal one c. 5 × longer than wide at maturity, greyish-green, to strongly anthocyanic at maturity, strongly laterally compressed, distinctly keeled, thin, keel to 3/4 the length and marginal veins and sometimes the intermediate veins to 1/2 the length, loosely sericious to villous, between veins sparsely to moderately densely appressed pubescent or occasionally glabrous on the proximal lemma, keel distally weekly scabrous, intermediate veins distinct, not extending to near the margin, margins inrolling below at maturity, very narrowly hyaline above, edges smooth or with a few hooks, apices acute, briefly hyaline; Paleas shorter than the lemmas by c. 0.5 mm, keels scabrous distally, sometimes weakly so, sparsely puberulent medially or nearly so, glabrous. **Flowers** chasmogamous; Lodicules c. 0.25 mm long, obscurely lobed; Anthers 1.2–1.4 mm long, vestigial in the upper floret. **Caryopsis** 1.8–2 mm long, strongly laterally compressed, sulcate, honey brown, firm, adherent to the lemma and palea, hilum 0.2 mm long, elliptical. 2*n* = unknown.

##### Distribution.

Endemic to the central Andes of Peru. Known from Huancavelica and Huánuco, although the Huánuco specimen is only tentatively placed.

##### Habitat.

Shrublands on Andean slopes at mid elevations.

##### Conservation status.

Data insufficient.

##### Additional specimens examined.

One other specimen appears to represent this species but is too immature to be certain. The specimen in question has extravaginal shoots branching from lower culm nodes; PERU: Región HUÁNUCO. Prov. Pachitea: Distr. Chaggla, canyon of the Rio Grande, c. 20 km above confluence with Rio Huallaga, E of Huánuco c. 44 air km, 1.7 air km SSW of Estación Huacachay (Huacachi), 2650 m, S9.86836 W75.83306, 8 Mar 2007, *Peterson*, *Soreng & Romaschenko 20369* (US!).

##### Discussion.

These plants bear similarities to *Poa aequatoriensis* but differ by having lemmas which are generally shorter (2.8–3.7 mm long), pubescent between the veins, and by more densely scabrous sheaths, with more-or-less smooth glabrous throats, ligules generally shorter. Tovar (1993) placed his voucher of this form (*2038*) in *Poa aequatoriensis*. However, among the 20 sheets and the US isotype reviewed of *Poa aequatoriensis*, all have lemmas that are smooth and glabrous between the veins (consistent with the description of Ecuadorian material by Hjorth, 1991), and the keel and marginal veins can be glabrous or sparsely puberulent. The species also bears some slight similarity to *Poa myriantha* Hack. and *Poa hieronymi* Hack. from the Yungas cloud-forests, Argentina, that differ by size of the anthers and ligule being much smaller (anthers <1 mm long, ligules <1 mm long) and overall habit being larger (culms 60–350 cm long with 10–15 internodes, and panicles 20–36 cm long) and glumes having both antrorse and retrorse scabrocities.

**Figure 2.**
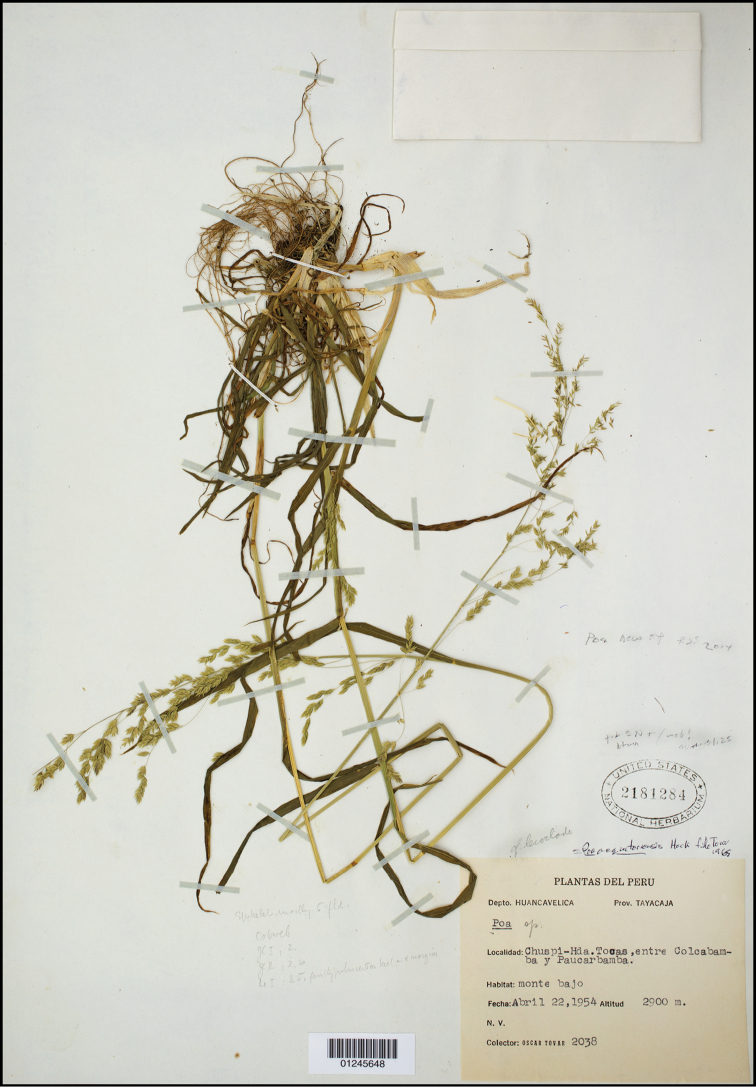
*Poa tayacajaensis*. Digitized holotype specimen *Ó. Tovar. S. 2038* (US-2181284).

**Figure 3.**
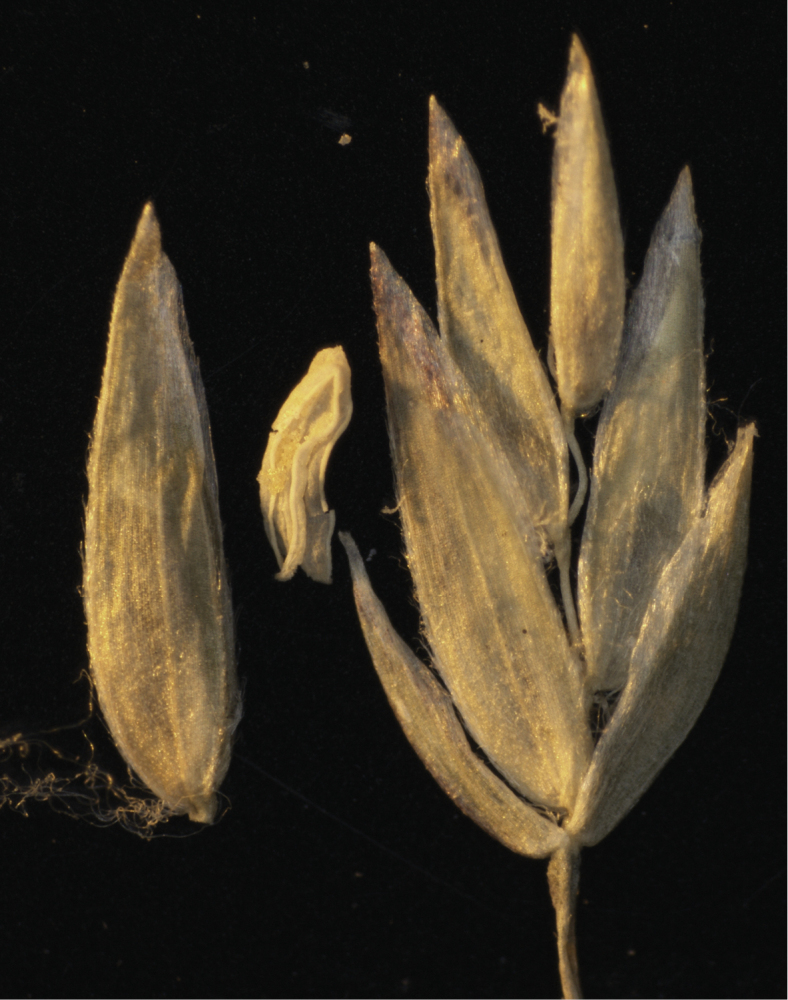
*Poa tayacajaensis*. Photograph of individual floret, anther, and spikelet (from top to bottom) of holotype specimen *Ó. Tovar. S. 2038* (US-2181284). (Photograph by R.J. Soreng).

#### Poa urubambensis

Taxon classificationPlantaePoalesPoaceae

S.P. Sylvester & Soreng sp. nov.

urn:lsid:ipni.org:names:77155740-1

Fig. 4

##### Type.

PERU. Región CUSCO. Prov. Calca: Distr. Calca, top of the prominent tower known by locals as “Kontorqayku”, 5 km NE of Huarán, 4401 m, S13°16'05.9", W72°01'17.2", 27 May 2011, *S.P. Sylvester 1317* (holotype: USM!; isotypes: CUZ!, K-000501720!, US!, Z-000099199!)

Plants gynomonoecious. **Perennials or (rarely) annuals**; Rhizomatous with well developed, slender, lateral tending, rhizomes, solitary and erect, or rarely tufted, often rooting from nodes. **Tillers** extravaginal. **Culms** (3–)15–30(–42) cm tall, decumbent to ascending, sometimes erect, fairly slender, not branching above the base, leafy; Culm nodes 1–2(–3), terete or slightly compressed, smooth, usually 1 node exposed at flowering; Culm internodes 3–15(–20) cm long, terete, smooth. **Leaves** mostly basal; Sheaths slightly compressed to keeled, smooth or scabrous along the veins; Butt sheaths papery, smooth, glabrous; Uppermost culm sheaths (3.5–)8–16 cm long, margins fused 25–60% the length, 1.8–2.7 × longer than their blades; Collars and throats smooth or scabrous, glabrous, collar margins of sterile shoot leaves sometimes flared; Ligules 1–4 mm long, not decurrent, scarious to hyaline, adaxially glabrous to scabrous, upper margins entire or irregularly dentate, apices acute and entire to irregularly dentate above, sterile shoot ligules generally shorter and more scabrous than those of the culm leaves; Cauline blades 2–15(–22) cm long, (1.5–)2–3 mm wide, flat or folded, margins often becoming involute, thin to moderately thin, soft or (rarely) curved, surfaces abaxially and adaxially lightly to moderately scabrous or rarely smooth, margins scabrous, narrowly to abruptly prow-tipped; Mid-cauline blades the longest, 10–22 cm long, shorter upward, flag leaf blade 3.5–7.7 cm long; Sterile shoot blades similar to cauline blades, sometimes more involute. **Panicles** (4–)8–13 cm long, erect, loosely contracted to open, ovoid to narrowly pyramidal, sparsely to moderately congested, with 13–40(–80) spikelets, proximal internode 1.4–3 cm long, smooth or scaberulous, usually scabrous towards its apex; Rachis with (1–)2–3(–5) branches per node; Primary branches ascending, fairly flexuous, weakly angled, moderately scabrous; Lateral pedicels mostly 3/4 to equaling the spikelets, moderately to densely scabrous, prickles moderately coarse; Longest branches 3–5 cm long, with 5–15 spikelets in distal 1/2, loosely arranged. **Spikelets** 3.7–6.5 mm long, to 3.7 × long as wide, lanceolate, laterally compressed, not bulbiferous, two toned; Florets 2–3(–4), proximal 1 or 2 florets hermaphroditic and distal 1 or 2 pistillate or sterile; Rachilla internodes terete, distal internodes 0.6–1 mm long, terete, smooth, glabrous; Glumes equal to subequal, narrow lanceolate, herbaceous and pale green below, scarious bronzy and sometimes anthocyanic in margins and apex, veins distinct, distinctly keeled, usually scabrous purely on the veins and sometimes between veins, margins scarious-hyaline, edges entire or dentate, smooth, apices acute, entire; Lower glumes 3.1–3.5 mm long, 2/3–4/5 as long as adjacent lemmas, 1-veined, narrow; Upper glumes 3.4–3.9 mm long, c. 2 × wider than the lower, 3-veined; Calluses glabrous; Lemmas (the lowest) 3.2–3.9 mm long, 5-veined, lanceolate in side-view, the proximal one c. 4–8 × longer than wide at maturity, proximally light green and distally bronzy-anthocyanic at maturity, moderately laterally compressed, thin, keeled, keels to 1/3–5/6 and marginal veins to 3/5–4/5, proximally smooth, keel and sides distally sparsely to moderately scaberulous, intermediate veins obscure to moderately prominent, not extending to near the margin, margins broadly scarious-hyaline, edges scabrous, apices acute; Paleas to 1.6 mm shorter than the lemma, glabrous, keels distally sparsely to moderately scabrous, between keels narrow (0.3–0.4 mm). **Flowers** chasmogamous; Lodicules c. 0.25 mm long, obscurely to shallowly lobed; Anthers 0.7–1.1(–1.3) mm long, infrequently vestigial in upper florets of spikelets. **Caryopses c.** 1.9 mm long, elliptical in side-view, sulcus broad and shallow, brown, hilum 0.2 mm long, oval, grain free from the palea. 2*n* = unknown.

##### Distribution.

Restricted to undisturbed areas of *Polylepis* woodland in hard to access areas throughout the Cordillera Urubamba, Cusco, Peru, at 4390–4802 m. Known from three localities; 1) Cliff ledges of the prominent SW facing cliff face 1.5 km S (170°) of Cancha Cancha village, Huarán. 2) Ledges of the prominent tower known by locals as “Kontorqayku”, 5 km NE of Huarán. 3) Ridgeline to the W of Laguna Manalloqsa, Área de Conservación Privada (ACP) Mantanay, 10 km up the valley from Yanahuara in the small valley 3 km E of Laguna Ipsaycocha.

##### Habitat.

Relatively dry and exposed sites in montane *Polylepis* forest and forest edges.

##### Etymology.

The name ‘urubambensis’ refers to the Cordillera Urubamba.

##### Conservation status.

This narrow endemic is locally common.

##### Additional specimens examined.

PERU. Región CUSCO. Prov. Calca: Distr. Calca, large ledge situated on the prominent SW facing cliff face 1.5 km S (170°) of Cancha Cancha village, Huarán, 4524 m, S13°14'35.1", W72°01'14.1", 21 March 2011, *S.P. Sylvester 812* (CUZ!, MO!, US!, Z!); Distr. Calca, large ledge situated on the prominent SW facing cliff face 1.5 km S (170°) of Cancha Cancha village, Huarán, 4517 m, S13°14'35.0", W72°01'13.7", 24 March 2011, *S.P. Sylvester 869* (CUZ!, US!, Z!); Distr. Calca, within the SW facing forest at the top of the prominent tower known by locals as “Kontorqayku”, 5 km NE of Huarán, 4390 m, S13°16'07.7", W72°01'16.8", 11 June 2012, *S.P. Sylvester 1636* (US!); Distr. Calca, within the SW facing forest at the top of the prominent tower known by locals as “Kontorqayku”, 5 km NE of Huarán, 4390 m, S13°16'07.7", W72°01'16.8", 11 June 2012, *S.P. Sylvester 1637* (CUZ!, SI!, US!, Z!); Distr. Calca, top of the prominent tower known by locals as “Kontorqayku”, 5 km NE of Huarán, 4401 m, S13°16'05.9", W72°01'17.2", 11 June 2012, *S.P. Sylvester 1695* (CUZ!, US!, Z!); Prov. Urubamba: Distr. Urubamba, ACP Mantanay, 10 km up the valley from Yanahuara in the small valley 3 km E of Laguna Ipsaycocha, ledges on cliff side 250°W of Laguna Manalloqsa, 4676 m, S13°12'01.3", W72°08'47.4", 28 January 2011, *S.P. Sylvester 403* (CUZ!, US!); Distr. Urubamba, ACP Mantanay, 10 km up the valley from Yanahuara in the small valley 3 km E of Laguna Ipsaycocha, topmost of the ridge to the W of Laguna Manalloqsa, 4802 m, S13°12'08.9", W72°08'43.9", 25 June 2012, *S.P. Sylvester 1727* (CUZ!, MO!, US!, Z!).

##### Discussion.

This new species is similar to other members of Poa sect. Homalopoa s.l. from Peru, Bolivia and Argentina, all of which have open panicles and spikelets with 2–5(–8) florets, the lowermost florets hermaphroditic while the upper florets are pistillate. *Poa urubambensis* is easily recognised in the field by the combination of an open-panicled inflorescence, glabrous lemmas and calluses, and exceptionally small anthers for members of Poa supersect. Homalopoa. *Poa urubambensis* also bears resemblance to *Poa oscariana*, but is distinguished by a less robust habit, the leaf blades being mainly basal and the anthers being smaller.

This species was found during a large scale ecological study attempting to reconstruct the potential natural vegetation (PNV) and soils of the high-elevation Puna grasslands (see Heitkamp et al. 2014 and Sylvester et al. 2014 for pilot studies). In this research, pristine zonal vegetation, only accessible with mountaineering equipment, was compared with surrounding slopes which have been grazed and burnt consistently over millennia (Thompson et al. 1988; Chepstow-Lusty et al. 1996, 2009; Kuentz et al. 2011). *Poa urubambensis* was a common element in undisturbed Puna vegetation in the Cordillera Urubamba, being found associated with *Polylepis* Ruiz & Pav. forests from three different sites and also found growing alongside other species new to science, e.g. *Bartsia lydiae* S.P. Sylvester (2014: 41). Following indicator species analyses, *Poa urubambensis* has been found as an indicator species for the PNV, due to its frequency and abundance within relict patches of near natural vegetation (Sylvester et al., *unpubl. data*). This species has not been found in accessible, disturbed or secondary, vegetation at similar or lower elevations in the Andes of the Cuzco region, despite a more thorough botanical exploration. This may relate to its susceptibility to disturbance from grazing and burning (Sylvester, *pers. observation*).

**Figure 4.**
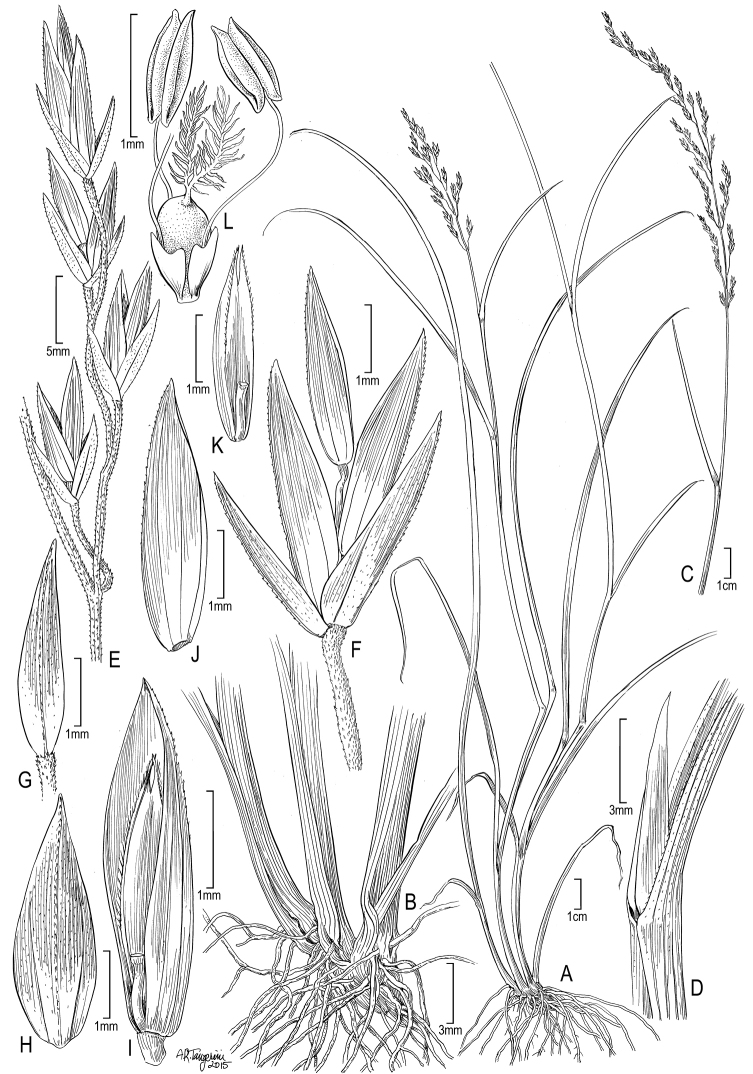
*Poa urubambensis*. **A** Habit **B** Basal portion of plant showing extravaginal shoots **C** Inflorescence **D** Ligular zone **E** Segment of inflorescence **F** Spikelet **G** Lower glume dorsal view **H** Upper glume dorsal view **I** Proximal floret **J** Lemma, lateral view **K** Palea, ventral view **L** Pistil, stamens and lodicules from perfect floret. Drawn from type material (*S.P. Sylvester 1317*, US).

### Species excluded

***Poa androgyna*** Pilg. **Ref**: Renvoize (1998: 144). **Discussion**: *Poa androgyna* has been described for Chile and Bolivia (Renvoize 1998; Soreng et al. 2003, 2016) with Renvoize (1998) mentioning the species to occur in Peru but not citing specimens and none have been encountered so far. The name is difficult to apply and taxonomists are still unsure of what it is exactly. Past authors (Hitchcock 1927; Standley 1936; Foster 1958; Brako and Zarucchi 1993) have placed this name as a synonym of *Poa horridula*, which is quite likely, but we are not certain. Plants identified as *Poa androgyna* have narrower leaf blades and more reflexed panicle branches than that typical of *Poa horridula*.

***Poa bromoides*** Vahl = ***Eragrostis bromoides*** (Vahl) Steud. **Ref**: Steudel (1854: 276). **Discussion**: Indiscrepancy centers around Brako and Zarucchi’s (1993) inclusion of *Poa bromoides* for Peru. As the type protologue of *Poa bromoides* states the plant to be cultivated: “Cult. Habitat (L)imae? Ex horto parisino habui” (Vahl 1794: 10), this effectively rules out the specimen being placed as *Poa*. Steudel’s (1854) description also includes “spiculis lanceolatis 25-floris glabris” (pp. 276), i.e. spikelets lanceolate, 25-flowered, that places further doubt on the specimen belonging to *Poa*. Grassbase (Clayton et al. 2006 onwards) accepts *Poa bromoides* Vahl as *Eragrostis bromoides* (Vahl) Steud. {appl. uncert. but valid}. Soreng et al. (2016), also accept it as *Eragrostis bromoides*.

***Poa lilloi*** Hack. **Ref**: Tovar (1965: 32; 1993: 127); Giussani et al. (2012: 319). **Ill**: Negritto and Antón (2000: fig. 11); Giussani et al. (2012: sp. 36, p. 319). **Discussion**: Specimens of *Poa lilloi* cited by Tovar (1993) to occur in Peru, *Ó. Tovar S. 2501* (US), *J.R. Swallen 7060b* (US) and *J.R. Swallen 7068* (US) have been redetermined as stunted versions of *Poa glaberrima*. The *J.R. Swallen 7060a* (US) specimen was redetermined as a stunted version of *Poa candamoana*. The specimens of *Poa lilloi* from JUNÍN cited by Brako and Zarucchi (1993) and Tovar (1993), *R. Ferreyra 5260* (US, USM) and *E. Cerrate 988* (USM[?]; Ill: Tovar 1965, lam. VIV, A.), have not been found. Certain specimens identified as *Poa lilloi* may actually be the new species *Poa ramoniana*. The type of *Poa ramoniana*, *R. Ferreyra 5200* (US, USM), was previously determined as *Poa lilloi*, and the USM isotype appears as a mixed collection of *Poa glaberrima* and *Poa ramoniana*. It is highly probable that all other specimens previously considered as *Poa lilloi* from Peru are stunted versions of either *Poa candamoana*, *Poa glaberrima* or *Poa kurtzii* that were collected from heavily grazed areas. Correctly determined specimens of *Poa lilloi* have been found from Bolivia, Chile and Argentina and occur in high elevation Puna grasslands above 3200 m. This species is most easily confused with *Poa kurtzii* from which it can be distinguished by being 7–20 cm tall, scabrous across the glume and lemma surfaces, lemmas 3–3.5 mm long, sometimes with a bit of pubescence on the lemma keel, sometimes a tiny web emerging from the dorsal side of the callus. *Poa kurtzii* is 20–70 cm tall, has smooth glumes and densely scabrous lemmas, lemmas 4–4.5 mm long. *Poa kurtzii* is also gynomonecious, while *Poa lilloi* is gynodioecious.

***Poa supina*** Schrader **Ref**: Tovar (1993: 126); Soreng 2007: 529. **Ill**: Soreng (2007: p. 529). **Discussion**: This taxon is highly unlikely to occur in South America with all specimens identified as *Poa supina*, so far encountered, pertaining to *Poa annua*. These include all specimens encountered from Ecuador and Bolivia (Simon Lægaard *pers. communication*). Tovar (1993) includes *Poa supina* in his treatment of Peru separating it from *Poa annua* and *Poa infirma* by having glabrous lemmas, or only lightly pubescent on the nerves, and swollen culms. However, the main distinction between *Poa supina* and *Poa annua*, aside from sparser lemma pubescence, is in the length of the anthers with *Poa supina* having anthers (1.2–)1.6–2(–2.5) mm while *Poa annua* has anthers 0.7–1(–1.2) mm. Specimens examined of *Poa supina* from Peru (*J. Espinoza 2* [US]) were redetermined as *Poa annua*, but the voucher collections *Tovar & Rivas Martinez 7720* and *Tovar 7855* accepted by Tovar (1993) have not been seen by us. Also see note under *Poa infirma* about the origin of *Poa annua*.

## Supplementary Material

XML Treatment for Poa ramoniana

XML Treatment for Poa tayacajaensis

XML Treatment for Poa urubambensis

## Supplementary materials

Supplementary material 1 Supplementary key to the open-panicled species of *Poa* in Peru Data type: Adobe PDF file Explanation note: This morphological key emphasizes lemma indumentum. This dataset is made available under the Open Database License (http://opendatacommons.org/licenses/odbl/1.0/). The Open Database License (ODbL) is a license agreement intended to allow users to freely share, modify, and use this Dataset while maintaining this same freedom for others, provided that the original source and author(s) are credited.Steven P. Sylvester, Robert J. Soreng, Paul M. Peterson, Mitsy D.P.V. Sylvester
