# Supplementary material for: An updated checklist and key to the open-panicled species of Poa L. (Poaceae) in Peru including three new species, Poa ramoniana, Poa tayacajaensis, and Poa urubambensis
Source: PhytoKeys. 2016 Jun 30;(65):57–90. doi: 10.3897/phytokeys.65.7024 (PMC4957023; doi:10.3897/phytokeys.65.7024)
Supplement: Supplementary material 1 — Supplementary key to the open-panicled species of Poa in Peru [file phytokeys-065-057-s001.pdf]

<u>Supplementary Material 1. SUPPLEMENTARY KEY TO THE OPEN-PANICLED SPECIES OF *POA* IN PERU</u> Supporting Information to the paper Sylvester, S.P., Soreng, R.J., Peterson, P.M. & Sylvester, M.D.P.V. An updated checklist and key to the open-panicled species of *Poa* L. (Poaceae) in Peru including three new species, *Poa ramoniana*, *Poa tayacajaensis*, and *Poa urubambensis*. *Phytokeys* (2016).
Explanation note: This morphological key emphasizes lemma indumentum.

1. Lemmas with bifid apexes that are mucronate to short-awned; spikelets glabrous, with long rachillas 1.2–4.2 mm long; glumes short, less than ½ the length of the florets, sometimes absent..**2**
– Lemmas with obtuse to acute apexes, never bifid, mucronate or short awned; spikelets usually with some form of indumentum, less often glabrous, with short rachillas rarely longer than 1.2 mm; glumes less than half to equaling the length of the spikelet, never absent..........................**3**

2 (1). Glumes veinless, 0.1−0.5 (–0.7) mm long, minute or absent; plants straggling and stooling; culms 30–80(–100) cm tall ....***P. hitchcockiana***
– Glumes veined, 1−2 mm long, lower glume 1-veined, upper glume 3- or 4-veined; plants caespitose; culms 14−24 cm tall ....***P. sanchez-vegae***

3 (1). Glumes exceeding the florets; spikelets 2-flowered; lemmas 3 (rarely 5)-veined, glabrous, smooth or scaberulous; low tufted (sometimes rhizomatous in *P. trollii*) perennial plants mostly less than 10 cm tall; panicles 1–3.3 cm long **4**
– Glumes shorter than the proximal floret; spikelets 2–4(–6)-flowered; lemmas 5 (or 7)-veined, glabrous or pubescent, smooth or variously scabrous; annual or perennial plants of various habits, ranging mostly from 10–120 cm tall; panicles longer (sometimes short in the annuals)...............**6**

4 (3). Anthers 2–2.2 mm long in staminate specimens, vestigial in pistillate specimens; lemmas smooth throughout; plants sometimes rhizomatous ....***P. trollii***
– Anthers <1 mm long in lower floret of spikelets, sometimes vestigial in upper floret; lemmas scaberulous, at least on the keels (rarely smooth in *P. calycina*); plants densely tufted **5**

5 (4). Leaf blade abaxial surface shiny with veins indistinct....................................... ***P. calycina***
– Leaf blade abaxial surface dull with veins apparent...***P. swallenii***

6 (3). Culm nodes strongly compressed, lower culm nodes exposed; culms wiry; plants strongly rhizomatous with isolated shoots; all florets of spikelets hermaphroditic (sometimes anthers aborted late in development)..***P. compressa***
– Combination of characters not as above; culm nodes terete or slightly compressed, lower culm nodes usually held within sheaths; culms varying from wiry to robust; plants tufted or rhizomatous; upper floret(s) within spikelets sometimes pistillate, with rudimentary stamens (i.e. gynomonoecious) or all florets of spikelets hermaphroditic **7**

7 (6). Lemmas glabrous, smooth or scabrous (rarely sericeous at the base in *P. ramifera*); callus glabrous..**8**
– Lemmas, at least of the upper florets, pubescent or villous in their lower half (rarely glabrous in *P. annua*, scabrous-pubescent in *P. kurtzii*), or glabrous but then callus webbed, i.e. with long silky hairs emerging from below the lemma keel; callus glabrous or webbed **18**

8 (7). Culms erect, aerially branching well up the culm with lateral shoots that persist and flower in subsequent seasons .... ***P. ramifera***
– Culms not branching, or branching only near the base, or from decumbent culms..................**9**

9 (8). Summit of sheathes with prominent triangular auricles; spikelets 4–6-flowered ....***P. auriculata***
– Sheathes without auricles; spikelets 2–4(–5)-flowered **10**

10 (9). Lemmas surface completely smooth (sometimes distally obscurely to sparsely scaberulous in *P. ramoniana* but then plants 4–6 cm tall and ligules <1 mm long) **11**
– Lemmas slightly to strikingly scabrous between and on veins **14**

11 (10). Plants 4–6 cm tall; rhizomatous; ligules <1 mm long................................ ***P. ramoniana***
– Plants >10 cm tall; tufted or, if rhizomatous >100 cm tall; ligules 2–7 mm long **12**

12 (11). Leaf blades smooth throughout, (2–)4–9 cm long, 1–2(–2.5) mm wide when blade flattened.. ***P. glaberrima***
– Leaf blades densely scabrous throughout, 8–40 cm long, 3–10 mm wide when blade flattened...**13**

13 (12). Leaf blades conspicuously folded; plants 25–35 cm tall, tufted ....***P. gilgiana***
– Leaf blades flat; plants 100–150 cm tall, rhizomatous .... ***P. ayacuchensis***

14 (10). Anthers 0.7–1.1(–1.3) mm long; blades soft, thin and lax.................... ***P. urubambensis***
– Anthers 1.8–3.2 mm long; blades firm to soft, thin and lax **15**

15 (14). Leaf blades involute or the margins distinctly involute (rarely simply folded in *P. kurtzii*), densely scabrous (at least abaxially), firm to rigid; plants tufted; ligules (2.5–)5–15 mm long....**16**
– Leaf blades flat or folded, margins rarely distinctly involute, glabrous or lightly scabrous, lax or firm; plants erect (*P. fibrifera*) or rhizomatous (*P. oscariana*), sometimes tufted; ligules 1–7(–9) mm long **17**

16 (15). Ligules 8–15 mm long; panicles narrowly ovate, panicle branches ascending and subappressed, panicles included in the sheaths; lemmas scabrous ....***P. pearsonii***
– Ligules (2.5–)5–8 mm long; panicles amply ovate, panicle branches patent or reflexed, panicles exerted; lemmas scabrous or scabrous-pilose ....***P. kurtzii***

17 (15). Lower leaf sheaths often fibrous; ligules 1.5–5(–6) mm long; blades lax; spikelets 3–5-flowered, 5–9 mm long; anthers 2.4–3.5 mm long; rachilla internodes well exposed... ***P. fibrifera***
– Lower leaf sheaths not fibrous; ligules 6–9 mm long; blades somewhat firm; spikelets 2–3-flowered, 4.5–5 mm long; anthers 1.8-2 mm long; rachilla internodes short (compare with *P. gilgiana*) ....***P. oscariana***

18 (7). Plants annual; palea keels distinctly pubescent in part (very rarely glabrous) always without any hooks; callus glabrous...**19**
– Plants perennial; palea keels glabrous or pubescent in part, but always scabrous in part; callus glabrous or webbed, i.e. with long silky hairs (sometimes sparse) emerging from below the lemma keel (at least of the lower florets) **20**

19 (18). Anthers 0.2–0.5(–0.6) mm long; panicle branches ascending, spikelets usually crowded; foliage light green; plants ephemeral .... ***P. infirma***
– Anthers 0.6–1 mm long; panicle branches ascending to spreading, spikelets loosely arranged; foliage usually darker green; plants infrequently persisting for more than one season ...***P. annua***

20 (18). Callus glabrous; lemmas (at least the distal ones within a spikelet) softly villous-pubescent in their lower half **21**
– Callus webbed, i.e. with long silky hairs (sometimes sparse) emerging from below the lemma keel (at least of the lower florets); lemmas glabrous or distinctly to sparsely villous or serious pubescent along the keel and marginal veins only **24**

21 (20). Plants (30–)60–150 cm tall; leaf blades flat, sometimes folded towards their apices, usually more than 3 and up to 10 mm wide; inflorescence branches commonly verticillate; plants (sub-)rhizomatous with extravaginal shoots (if blades rather firm and folded but broad as in *P. horridula*, compare with *P. gilgiana*, possibly hybrids) ....***P. horridula***
– Plants usually <35 cm tall; leaf blades involute to narrowly convolute and 0.5–2 mm wide, or flat to folded and 1–5 mm wide in *P. grisebachii*; inflorescence branches solitary or paired (often 3 branches in basal nodes of *P. grisebachii);* plants usually with only intravaginal shoots.........**22**

22 (21). Leaf blade abaxial surface densely scabrous; ligules (2.5–)5–8 mm long, acute; lemmas scabrous-pilose; plants of semi-arid habitats ....***P. kurtzii***
– Leaf blade abaxial surface glabrous to scaberulous with prickles or hooks usually restricted to the leaf margin; ligules 0.5–3 mm long, truncate; lemmas pilose-villous towards base; plants of mesic or more arid habitats..**23**

23 (22). Leaf blades usually involute, apex narrowly but abruptly naviculate (prow-tipped); spikelets usually 3-flowered, (2.8–)4.3–5.5 mm long; culm basal sheath bases slightly inflated, shiny, and tough; plants of more mesic Puna, mostly 3700–4500 m ....***P. candamoana***
– Leaf blades flat or folded, somewhat lax, apex often tapered to a long slender point; spikelets 3–6-flowered, (5–)6–7.2 mm long; culm basal sheaths not as above; plants of more arid zones between 3000 and 4000 m (appears to hybridize with *P. kurtzii* where the two overlap)..***P. grisebachii***

24 (20). Leaf blades filiform or slightly broader, involute or sub-involute towards the base, 0.5–2 mm wide when expanded; lower lemma 3–4 (5?) mm long, glabrous; callus webbing the only indumentum present in the spikelet (web sometimes v. short and sparse, and present on basal florets only) .... ***P. pauciflora***
– Leaf blades flat or folded, usually >2 mm wide when expanded; lower lemma 2.3–6 mm long, variously glabrous or pubescent..**25**

25 (24). Basal sheaths glabrous and densely scabrous; lemmas smooth (or lightly scabrous near the apex), glabrous; callus webbing the only indumentum present in the spikelet..***P. scabrivaginata***
– Basal sheaths glabrous or lightly pubescent, smooth or lightly scabrous, or if densely scabrous then lemmas pubescent at least on the keel; lemmas smooth or scabrous, glabrous or pubescent in part **26**

26 (25). Upper ligules 0.9–2(–3) mm long, truncate; plants distinctly rhizomatous; lower sheaths smooth, sometimes lightly pubescent; lower lemma keel and marginal veins distinctly pubescent; spikelets with 2–6 florets; all florets of spikelets hermaphroditic (sometimes anthers aborted late in development)... ***P. pratensis***
– Combination of characters not as above; upper ligules 0.2–10 mm long, acute or rarely truncate; plants tufted (usually with short rhizomatous shoots in *P. huancavelicae* and prominent sub-rhizomatous extravaginal shoots present in *P. leioclada*); lower sheaths smooth to densely scabrous; lower lemma keel and marginal veins varying in indumentum from glabrous to

pubescent; spikelets with 2–3(–4) florets; upper floret within spikelets pistillate, with rudimentary stamens, or hermaphroditic (i.e. *P. trivialis*)..**27**

27 (26). Leaf blades folded, apex prominently naviculate (prow-tipped); plants weakly rhizomatous; lemma keels and marginal veins smooth or scaberulous, glabrous; callus webbing the only indumentum present in the spikelet .... ***P. huancavelicae***
– Leaf blades flat, apex not, or not prominently, naviculate; plants tufted, occasionally stooling and rooting at nodes (or with prominent sub-rhizomatous shoots present at the base in *P. leioclada*); lemma keels short pubescent in the lower ½, sometimes sparingly so on the marginal veins near the base (rarely glabrous in *P. aequatoriensis*); spikelet indumentum consisting of lemma pubescence (as mentioned above) and callus webbing..**28**

28 (27). Spikelets glomerate on branches; culm leaf ligules 1–2(–2.5) mm long, truncate or obtuse; short sub-rhizomatous shoots usually prominent at the base of the plant, these extravaginal, with brown cataphylls; spikelets 2–4-flowered; lemmas 2.5–3.5 mm long; sheaths smooth; leaf blades mostly folded, sometimes flat, firm; lowest floret of spikelets hermaphroditic, upper florets commonly pistillate ....***P.* cf. *leioclada***
– Spikelets diffuse throughout the panicle; culm leaf ligules 1–10 mm long, acute or rarely truncate; rhizomatous shoots absent, new shoots obscure, stoloniferous, extravaginal, with green cataphylls; spikelets 2–3-flowered; lemmas 2–4 mm long; sheaths smooth or scabrous; leaf blades mostly flat, flaccid; all florets of spikelets hermaphroditic (i.e. *P. trivialis*) or lowermost floret hermaphroditic and upper florets commonly pistillate (*P. tayacajaensis* & *P. aequatoriensis*)………………………..**29**

29 (28). Spikelet proximal lemmas pubescent on keel, lateral, and marginal veins; distal lemmas pubescent between the veins; sheaths densely scabrous; ligules 2–3.5 mm long .... ***P. tayacajaensis***
– Combination of characters not as above; spikelet proximal lemmas glabrous or sparingly pubescent on the keel, and sometimes marginal veins; distal lemmas often glabrous throughout; sheaths smooth to densely scabrous; ligules 1–10 mm long **30**

30 (29). Lower culm sheaths usually puberulent in the throat margins and/or along the collar margins, surfaces smooth to lightly scabrous; upper culm leaf ligules 1–5(–7) mm long, abaxially puberulous or scabrous; lowest floret of spikelets hermaphroditic, upper florets commonly pistillate; spikelets 3.5–5 mm long; lower lemma 3.6–4 mm long, intermediate veins faint to moderately pronounced; palea keels usually finely scabrous to some degree; anthers mostly 0.6–1.5 mm long .... ***P. aequatoriensis***
– Lower culm sheaths glabrous in the throat margins, surfaces nearly smooth to densely scabrous; upper culm leaf ligules 4–10 mm long, abaxially smooth or faintly scabrous; all florets of spikelets hermaphroditic; spikelets 2.3–3.5(–4) mm long; lower lemma 2.3–3(–3.5) mm long, intermediate veins distinctly pronounced; palea keels usually muriculate, sometimes minutely scabrous; anthers (1) 1.3–1.6 (1.8) mm long.. ***P. trivialis***
